# Supplementary figures and images for: Perturbations in eIF3 subunit stoichiometry alter expression of ribosomal proteins and key components of the MAPK signaling pathways
Source: eLife. 2024 Nov 4;13:RP95846. doi: 10.7554/eLife.95846 (PMC11534336; doi:10.7554/eLife.95846)

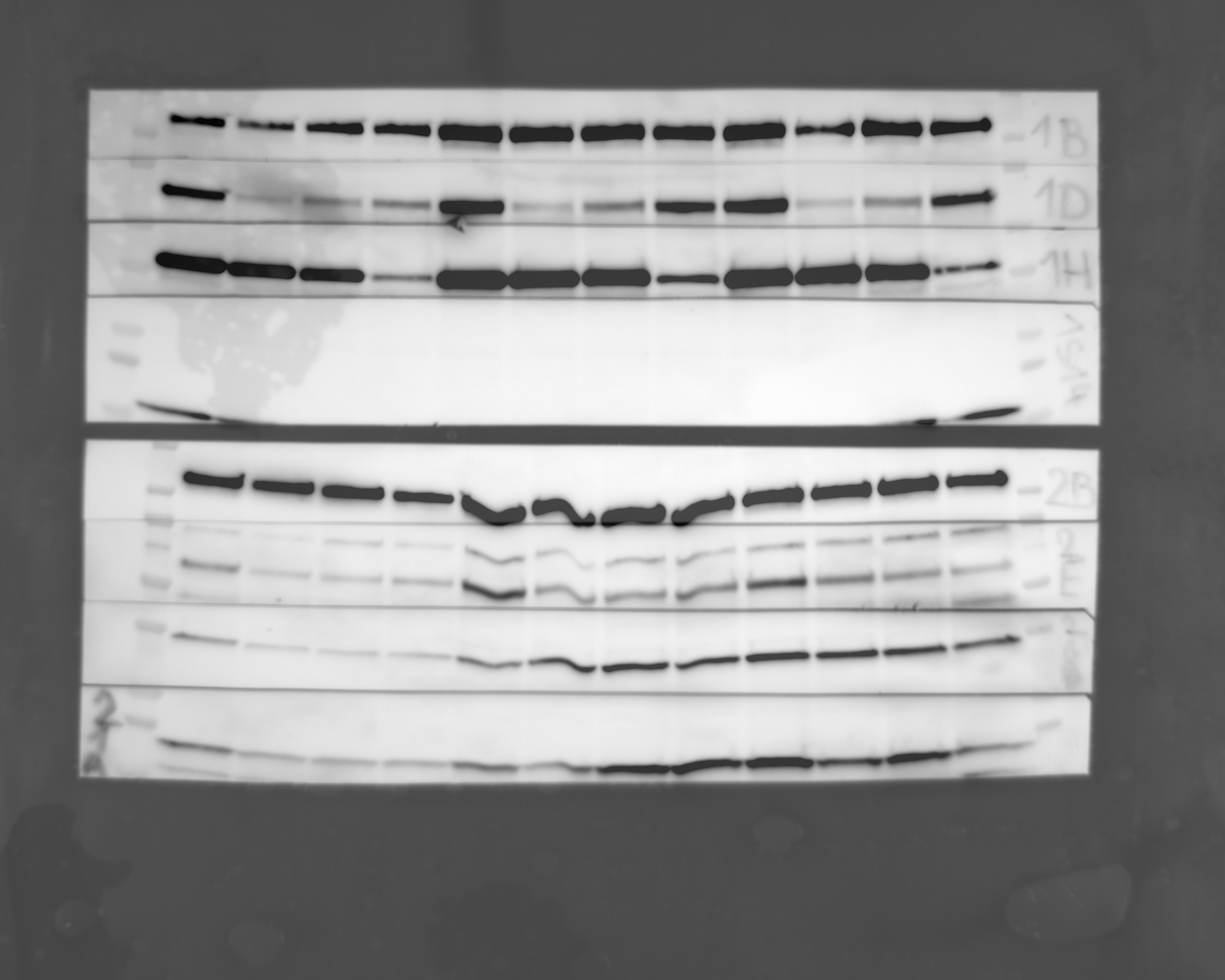

Supplement: Figure 1—figure supplement 1—source data 1. [file elife-95846-fig1-figsupp1-data1.zip › 2021-12-15-113113 eIF3B, D, H, GAPDH.tif]

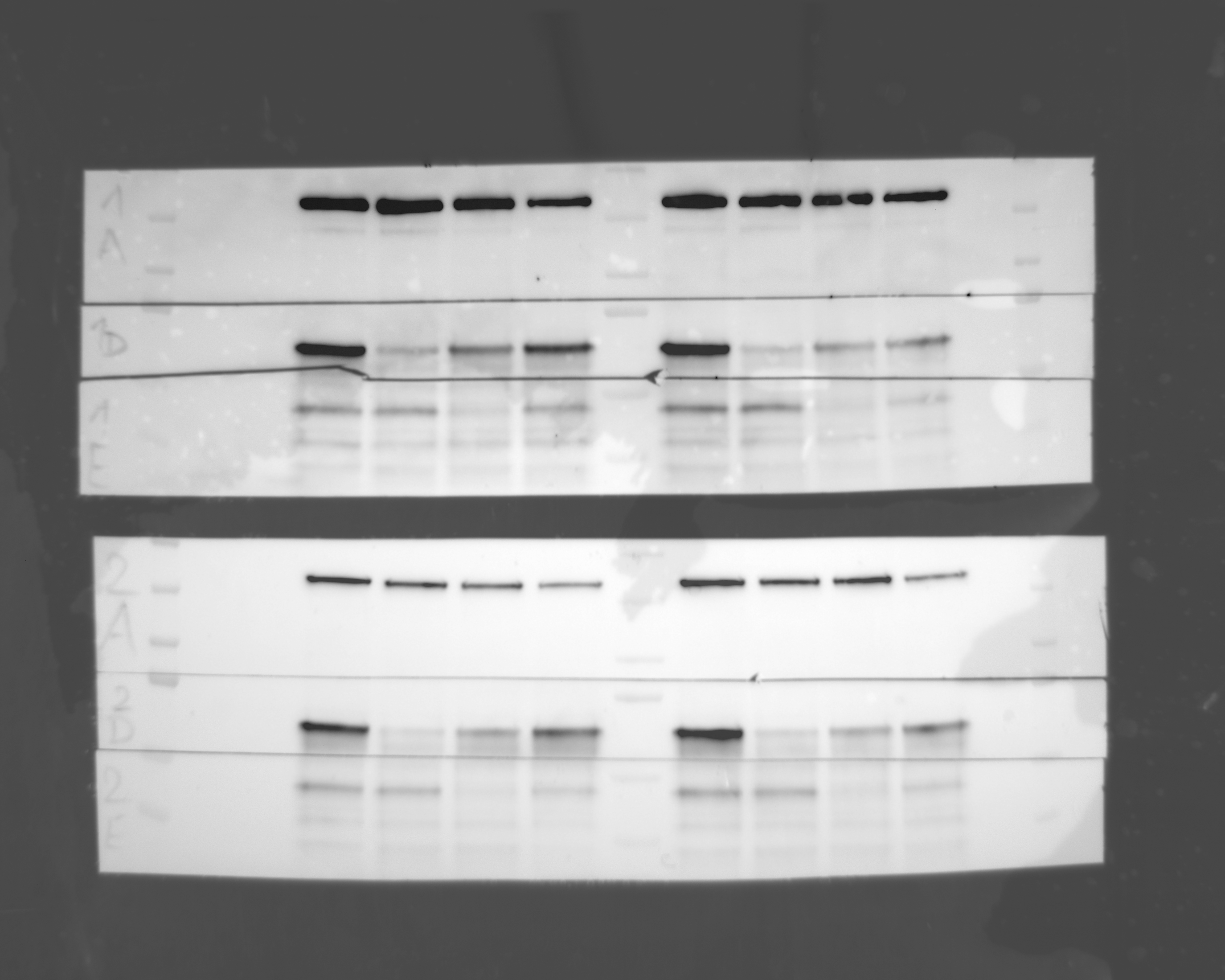

Supplement: Figure 1—figure supplement 1—source data 1. [file elife-95846-fig1-figsupp1-data1.zip › 2022-01-19-140050 eIF3E.tif]

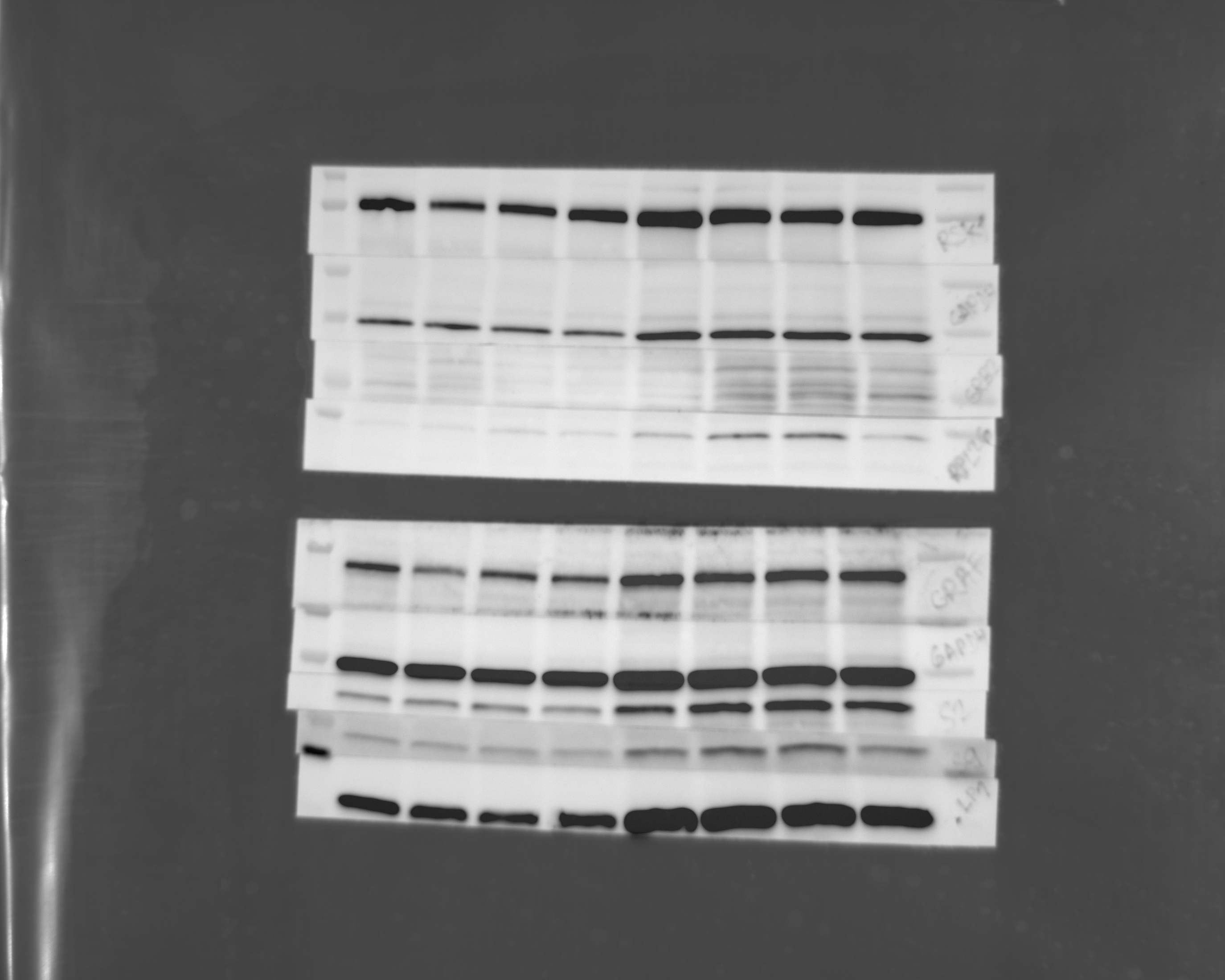

Supplement: Figure 3—source data 1. [file elife-95846-fig3-data1.zip › 2023-01-10-093201-RPS9, GAPDH, RPL26, GAPDH.tif]

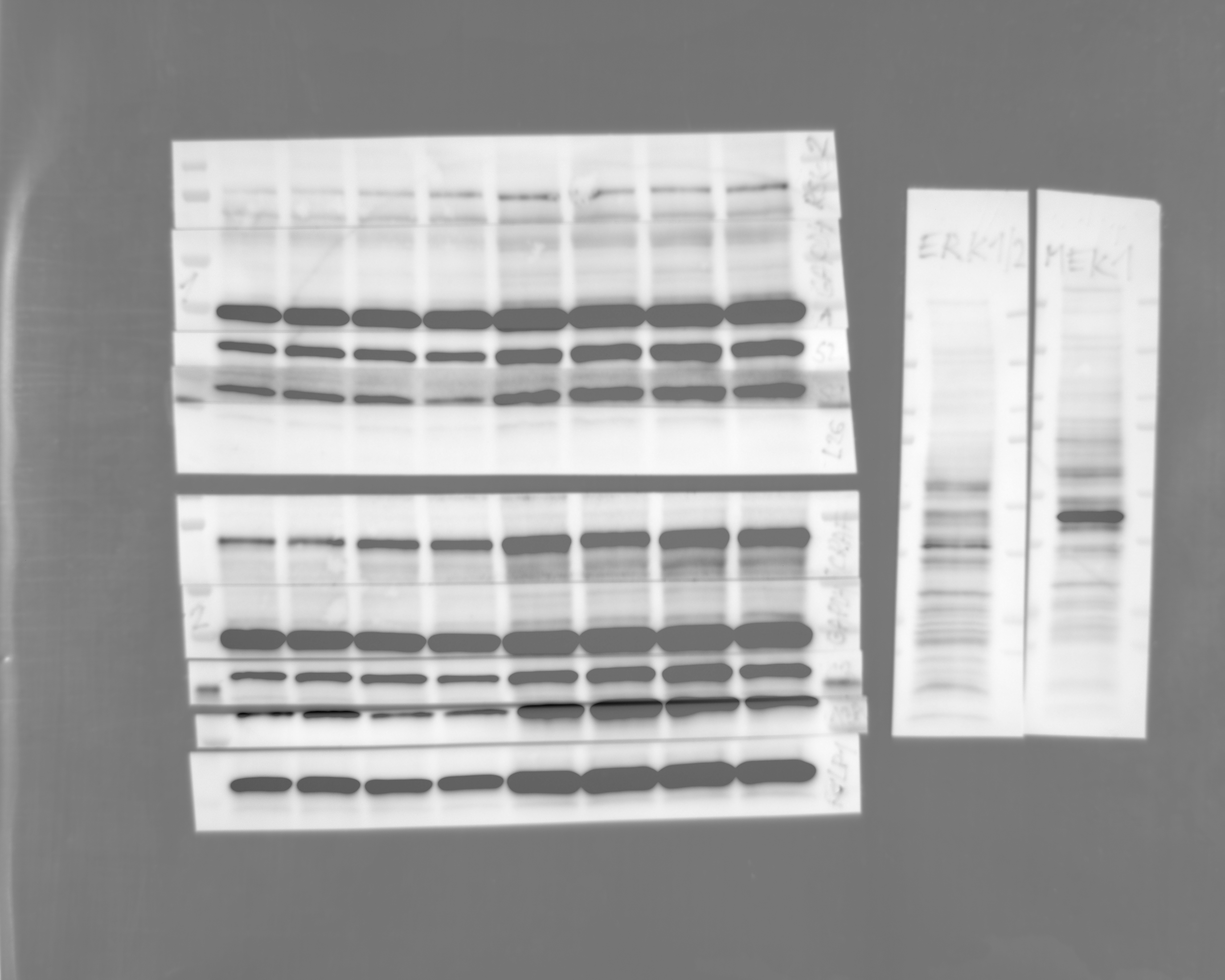

Supplement: Figure 3—source data 1. [file elife-95846-fig3-data1.zip › 2023-01-11-110719-RPS3, GAPDH.tif]

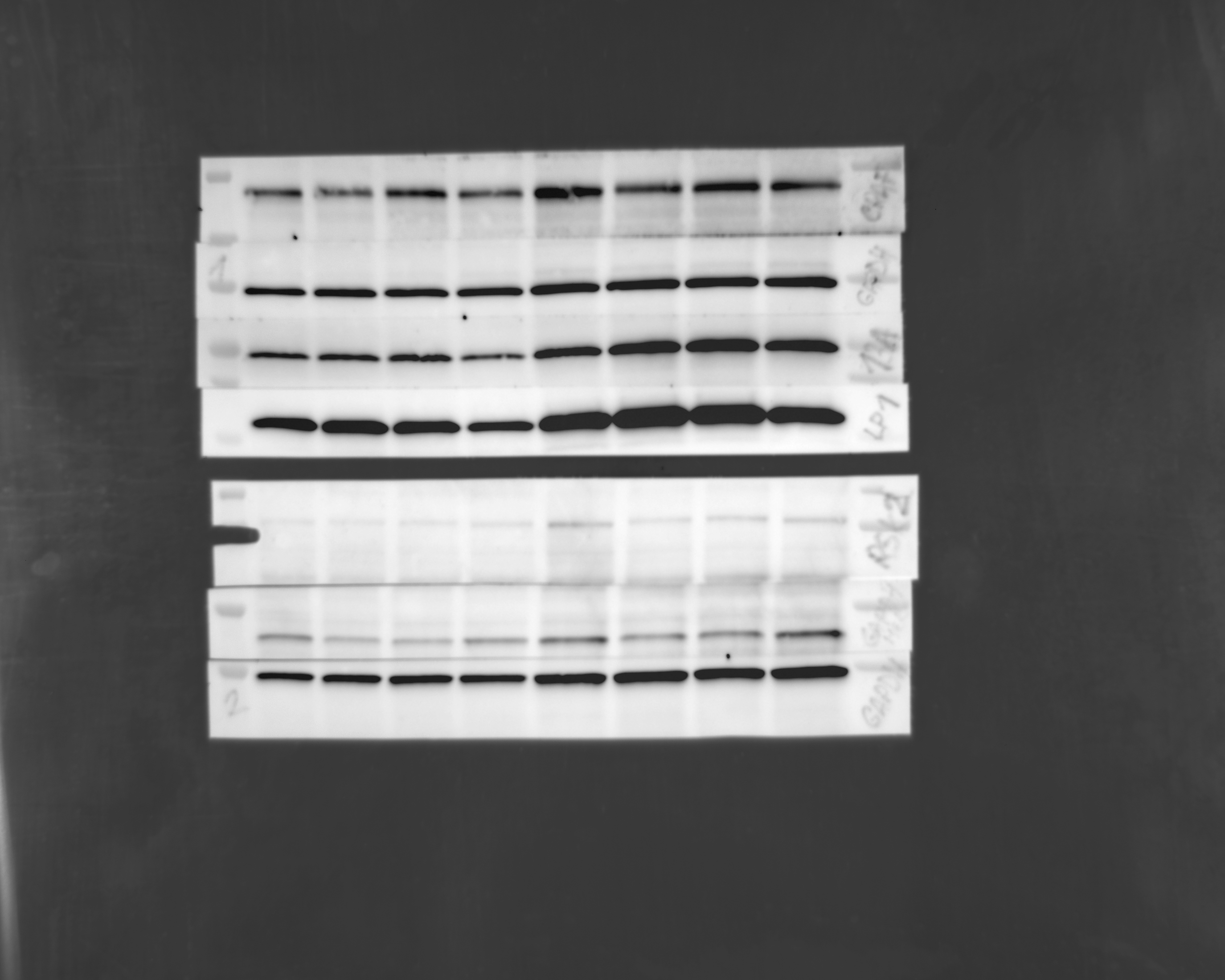

Supplement: Figure 3—source data 1. [file elife-95846-fig3-data1.zip › 2023-01-13-085557-RPL13A, GAPDH.tif]

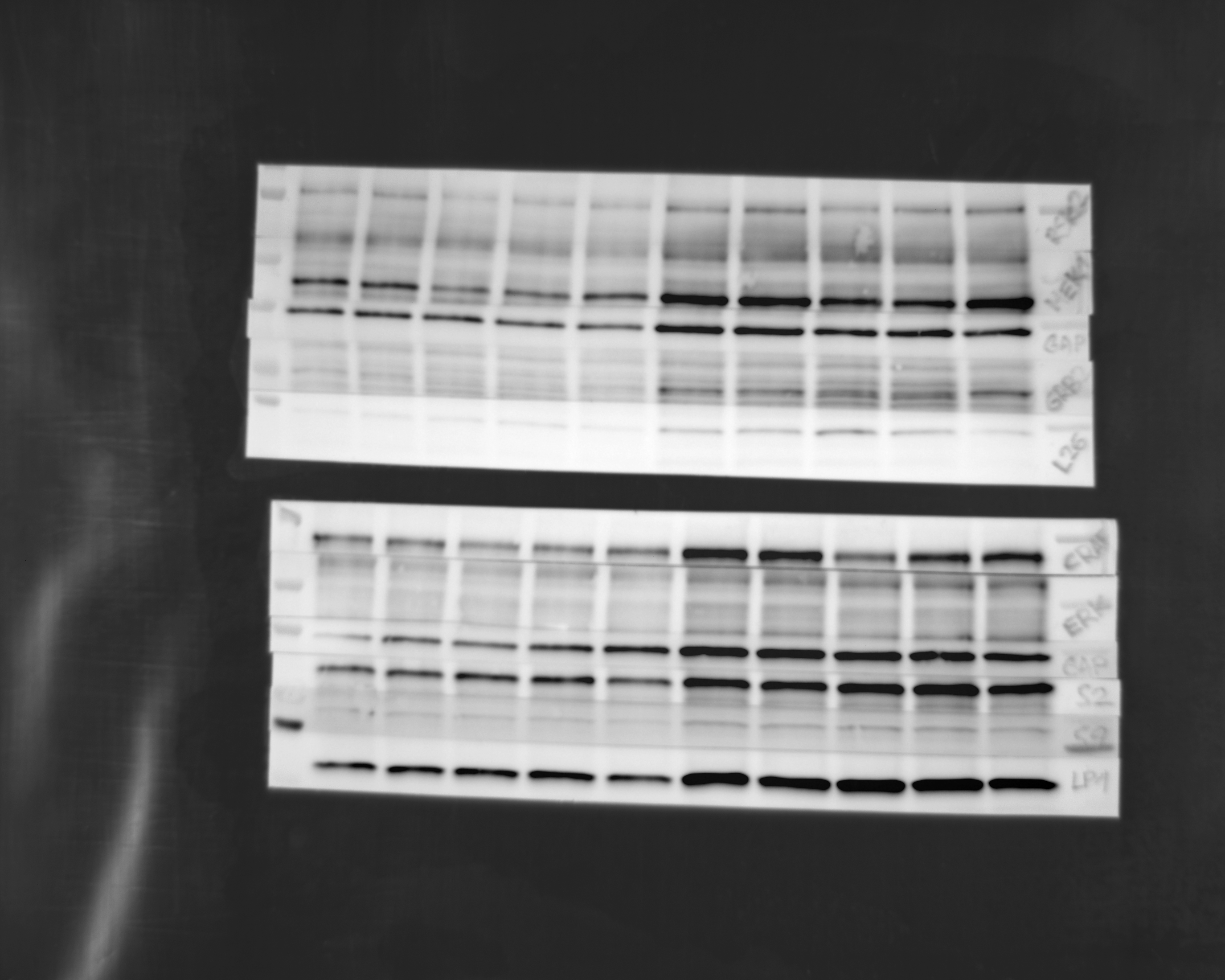

Supplement: Figure 3—source data 1. [file elife-95846-fig3-data1.zip › 2023-01-19-095620 RPS2, RPLP1, GAPDH.tif]

Figure 3E source data

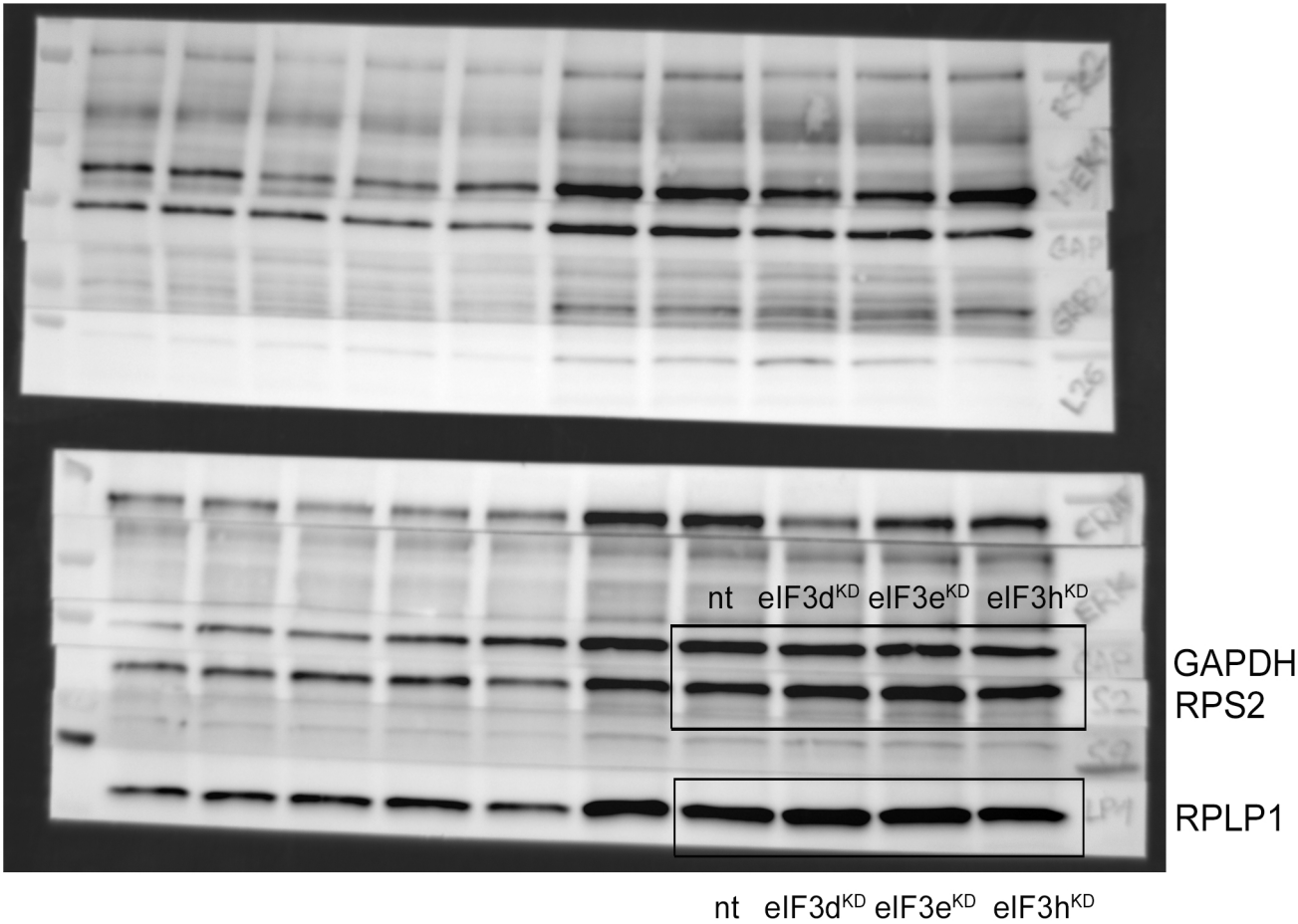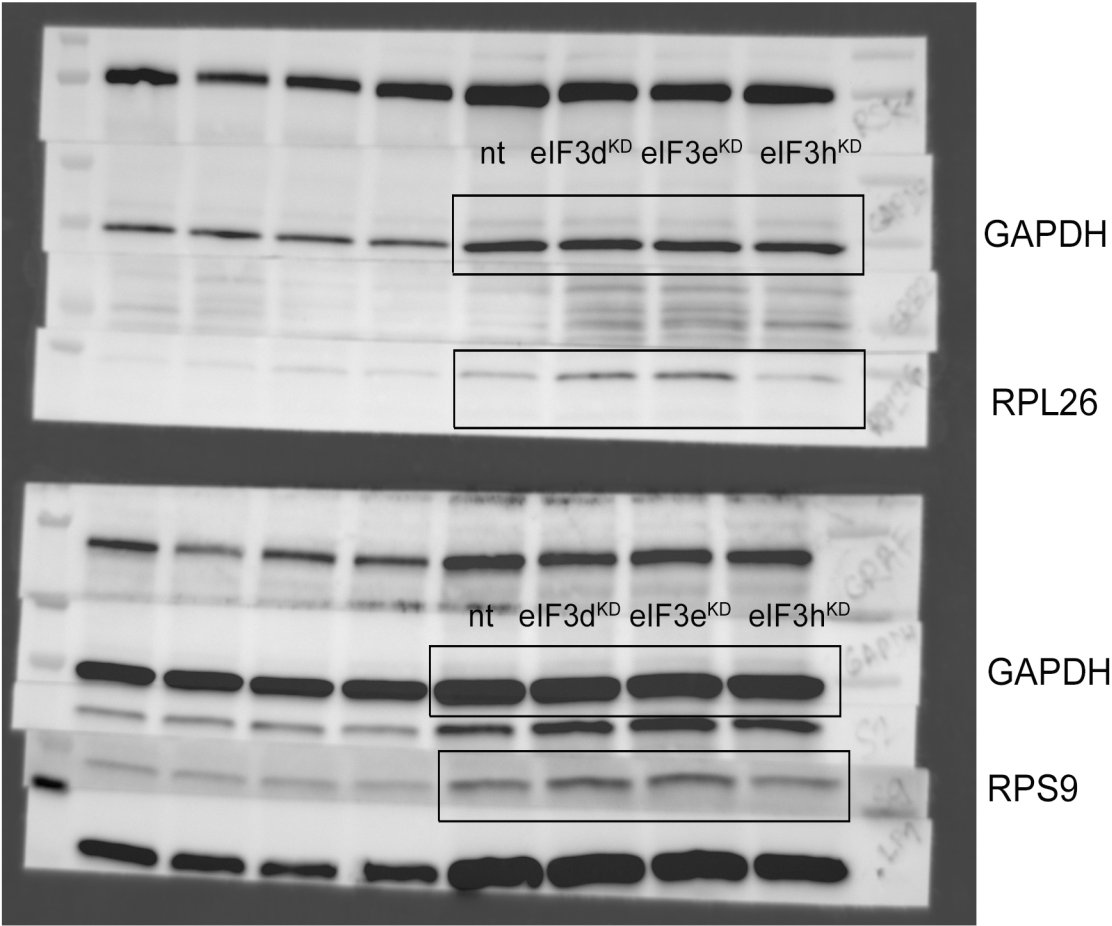

Figure 3E source data

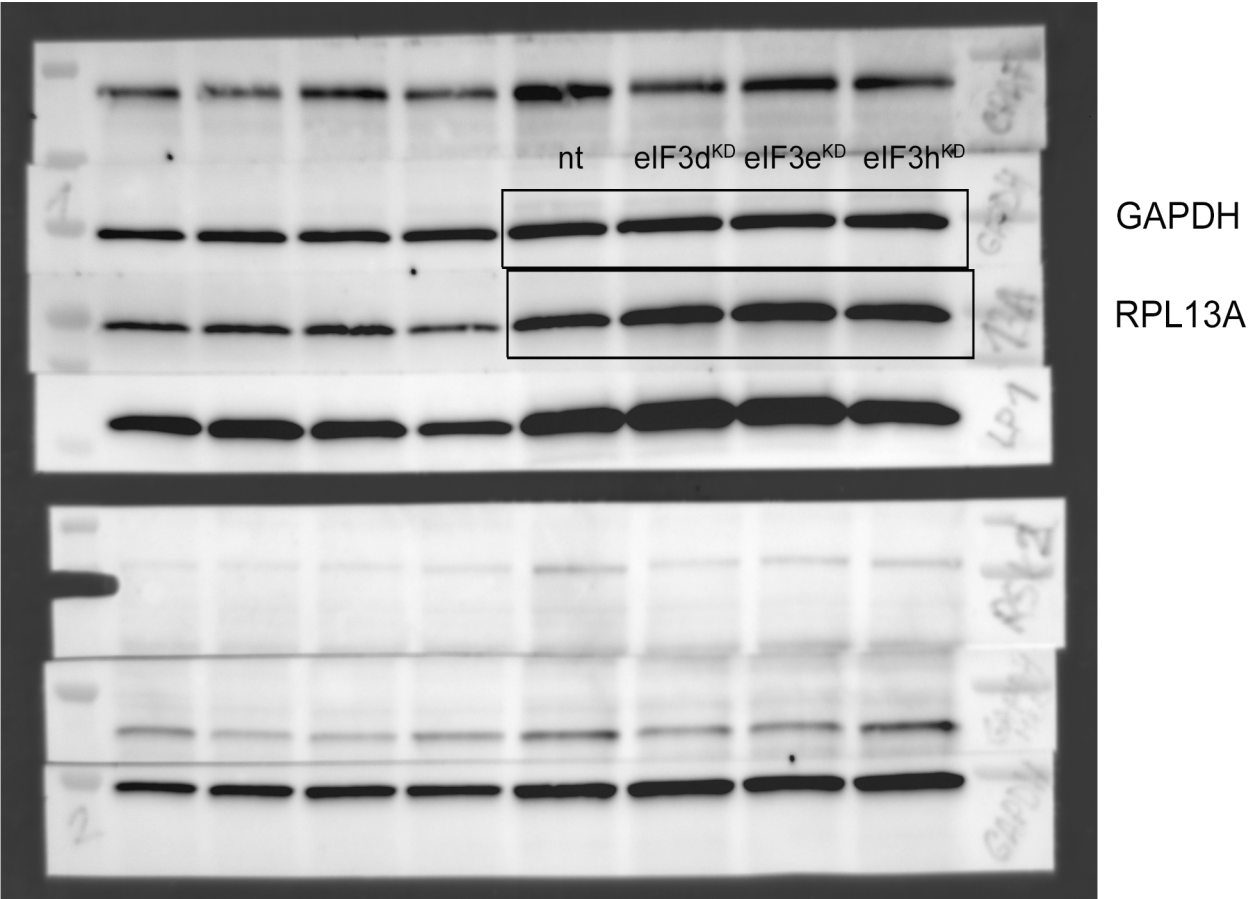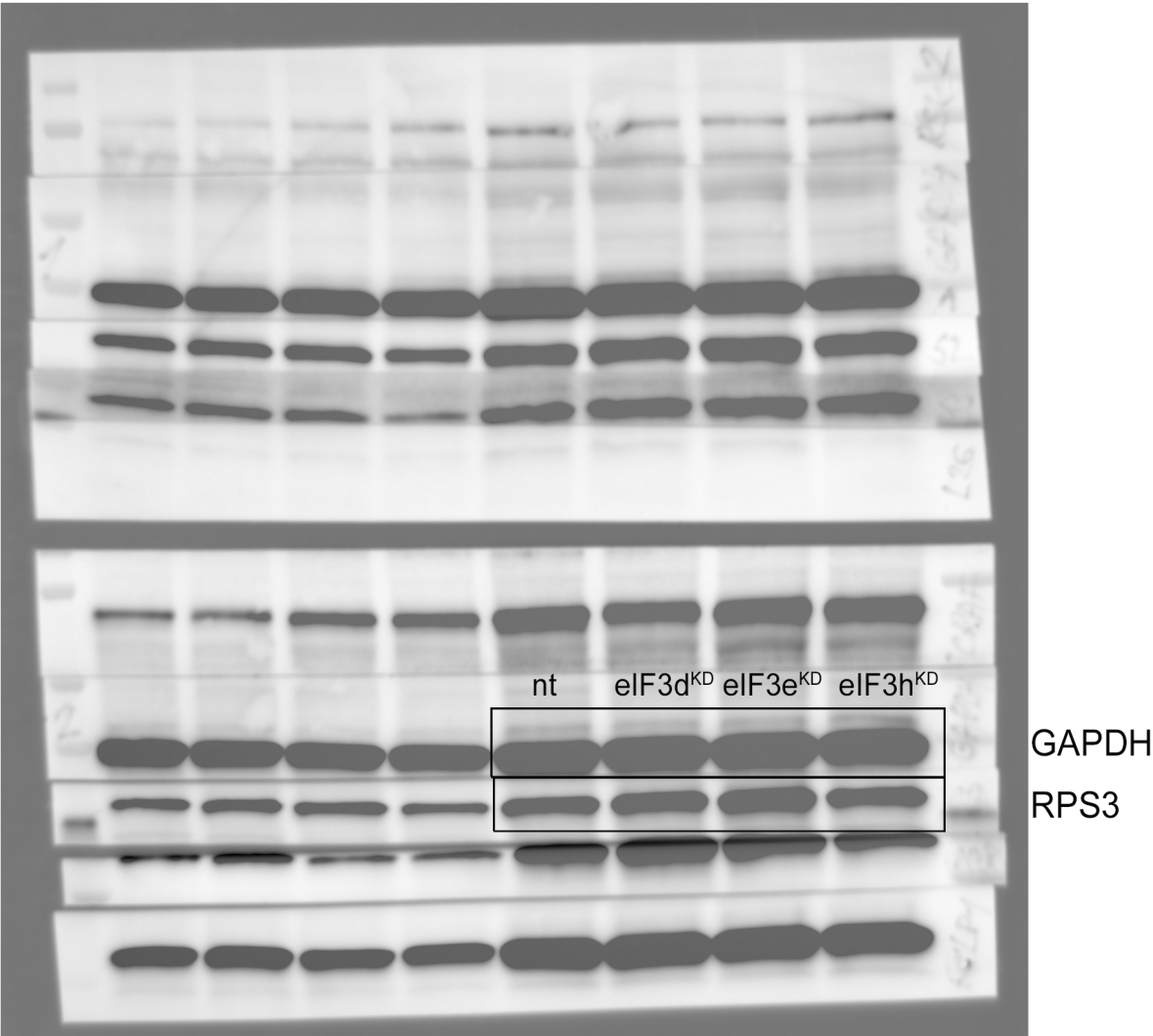

Supplement: Figure 3—source data 2. [file elife-95846-fig3-data2.pdf]

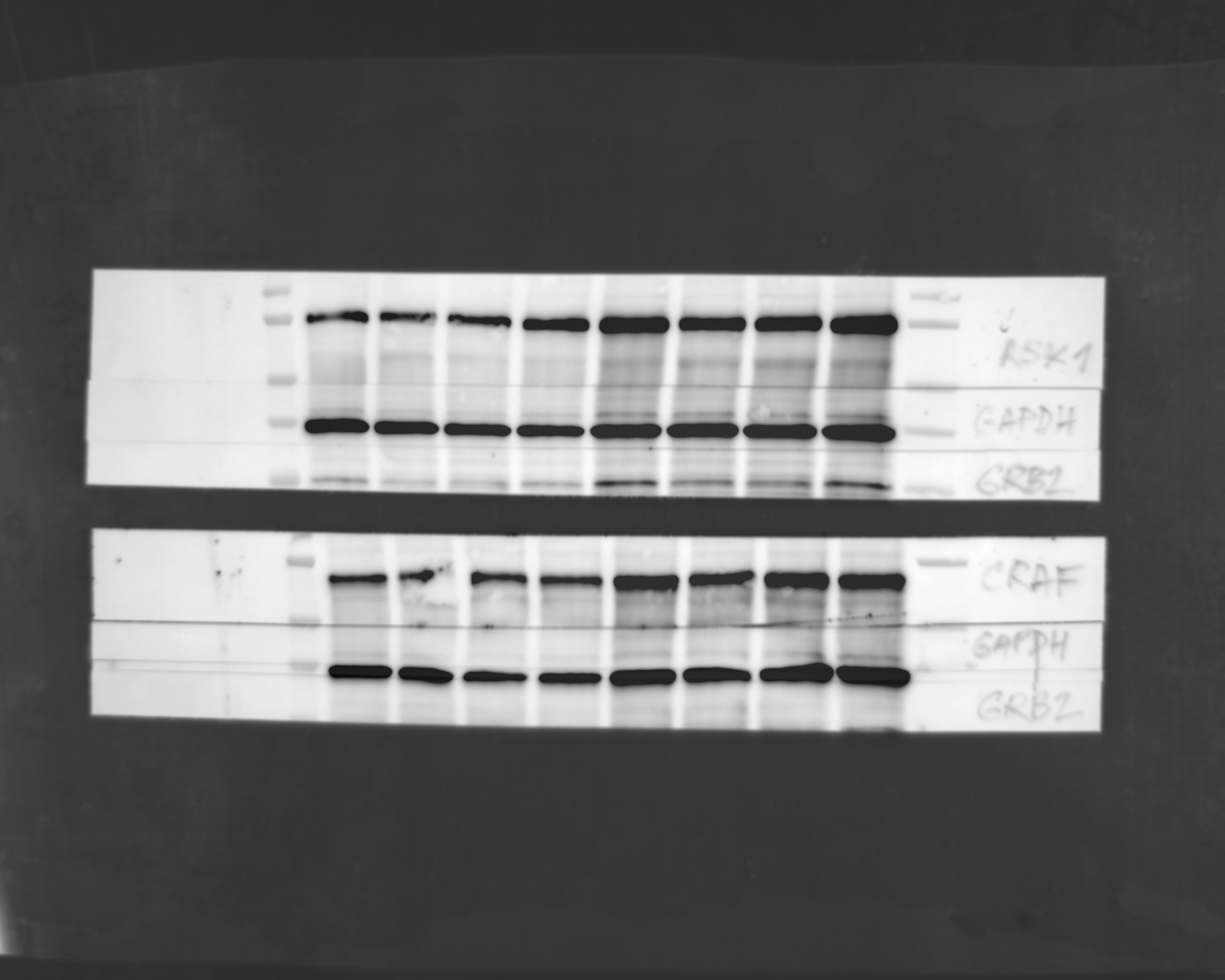

Supplement: Figure 5—source data 1. [file elife-95846-fig5-data1.zip › 5C/2023-01-06-101913 RSK1, GRB2, GAPDH.tif]

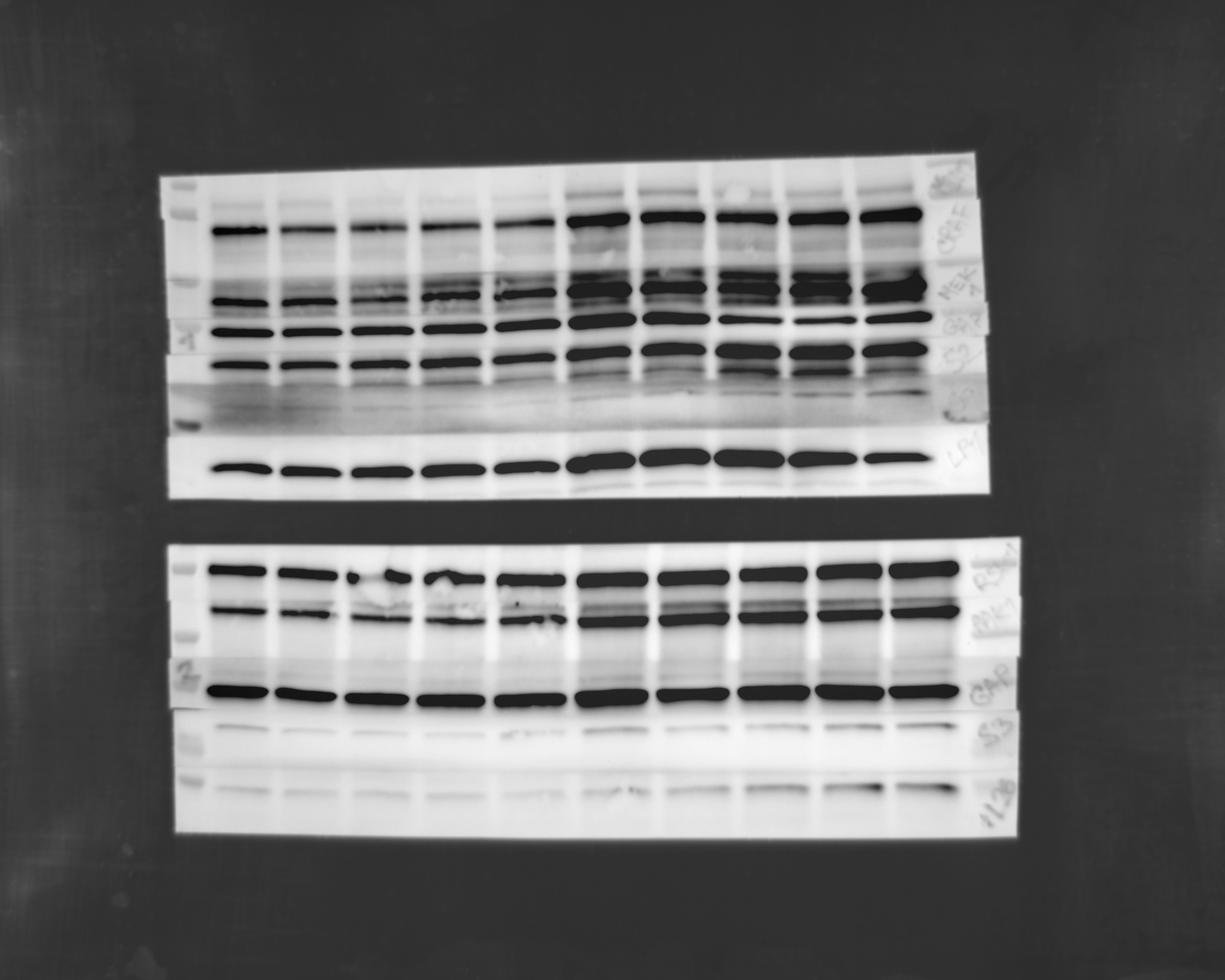

Supplement: Figure 5—source data 1. [file elife-95846-fig5-data1.zip › 5C/2023-01-25-130720 PAK1, GAPDH.tif]

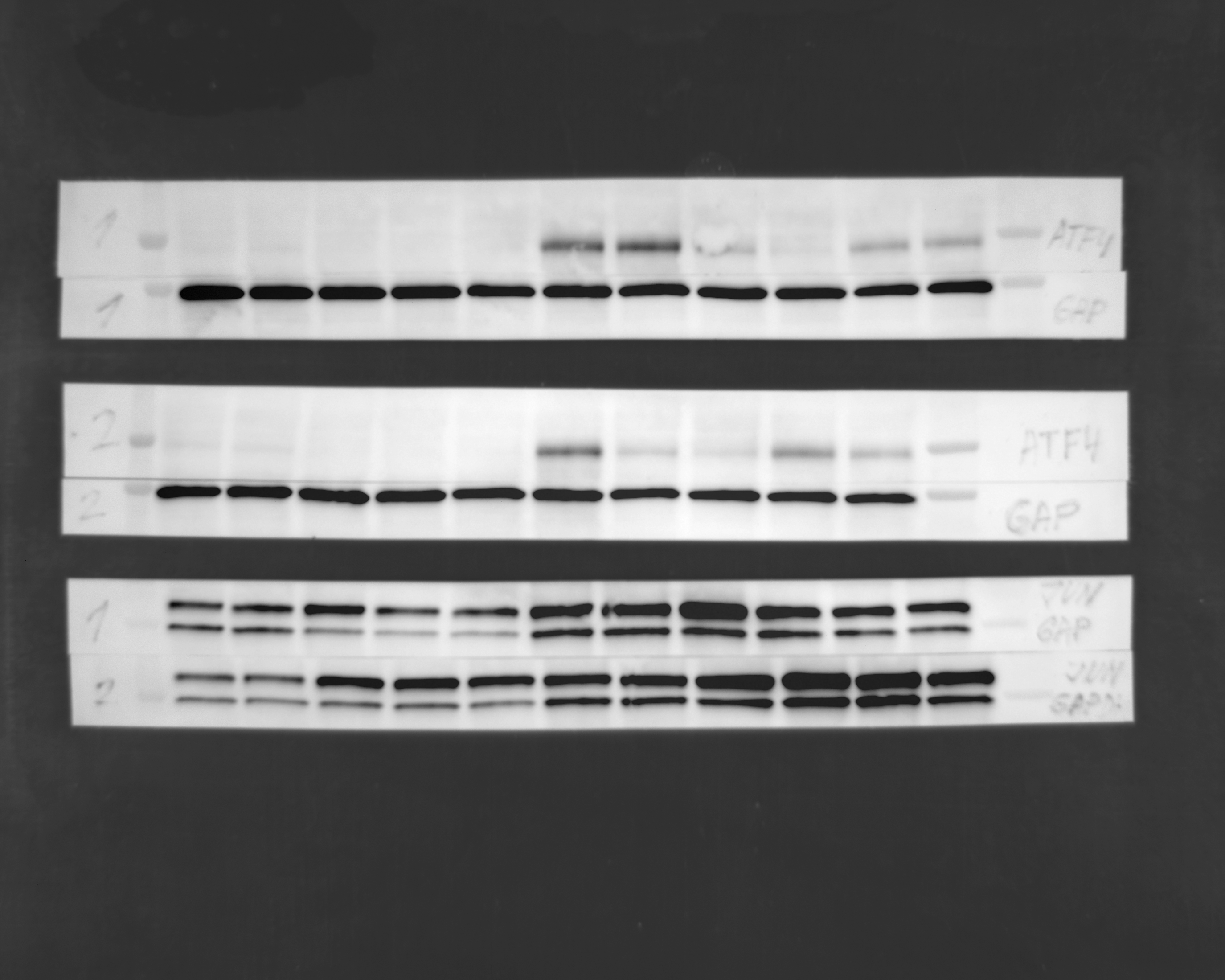

Supplement: Figure 5—source data 1. [file elife-95846-fig5-data1.zip › 5C/2023-02-15-114957 JUN, GAPDH.tif]

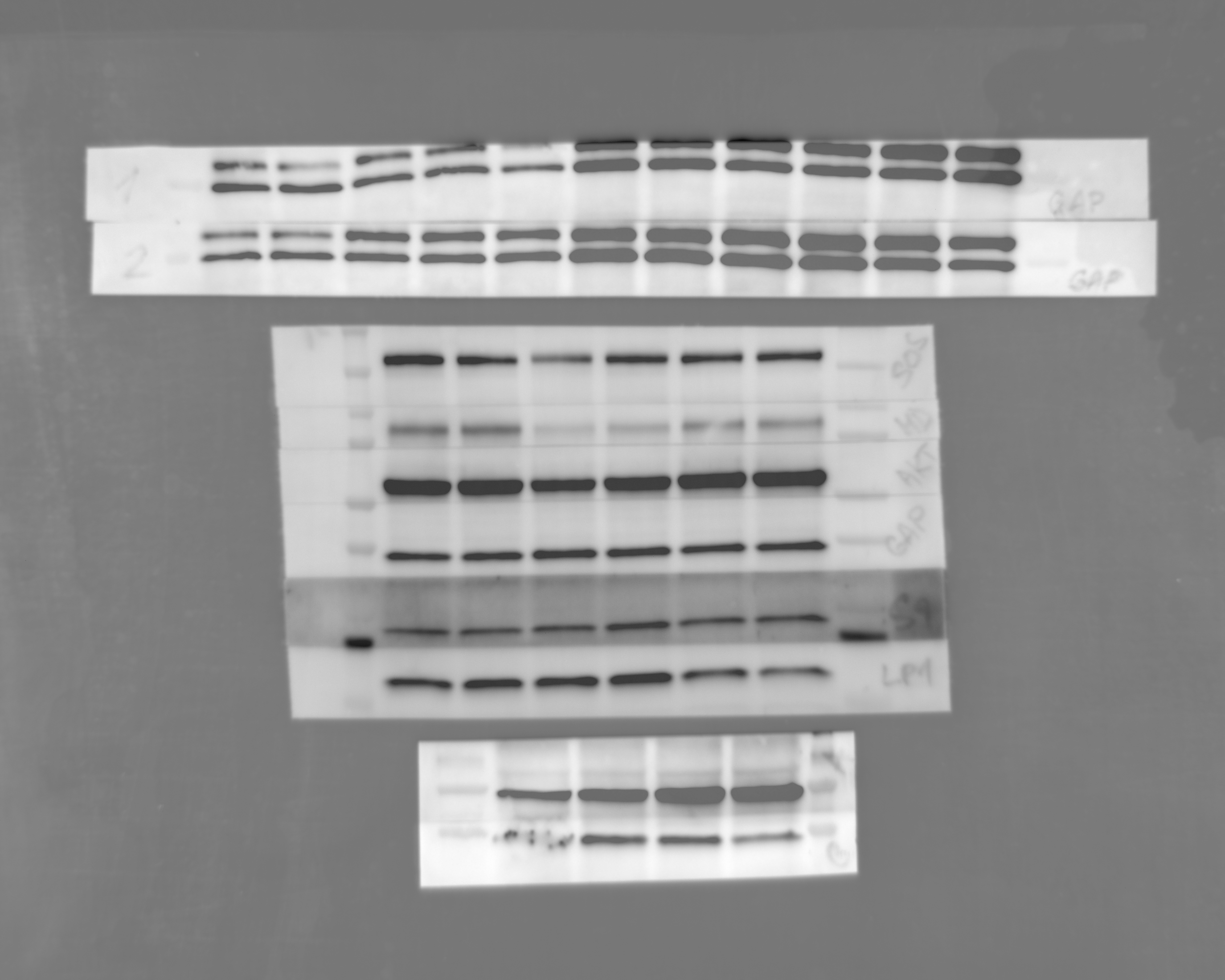

Supplement: Figure 5—source data 1. [file elife-95846-fig5-data1.zip › 5C/2023-02-23-091926 SOS1, AKT1, GAPDH.tif]

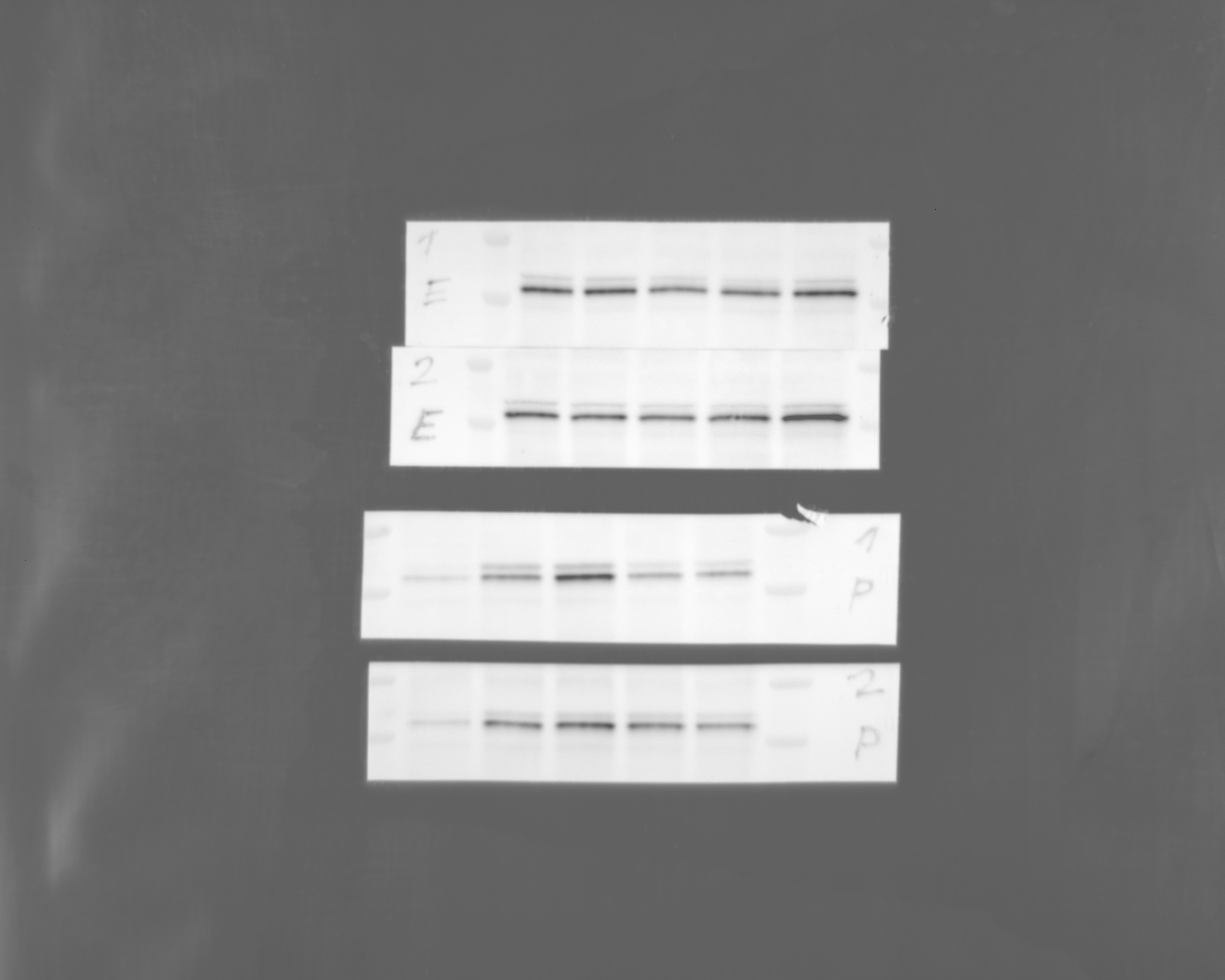

Supplement: Figure 5—source data 1. [file elife-95846-fig5-data1.zip › 5E/2023-05-03-112040 ERK12, Phospho ERK12.tif]

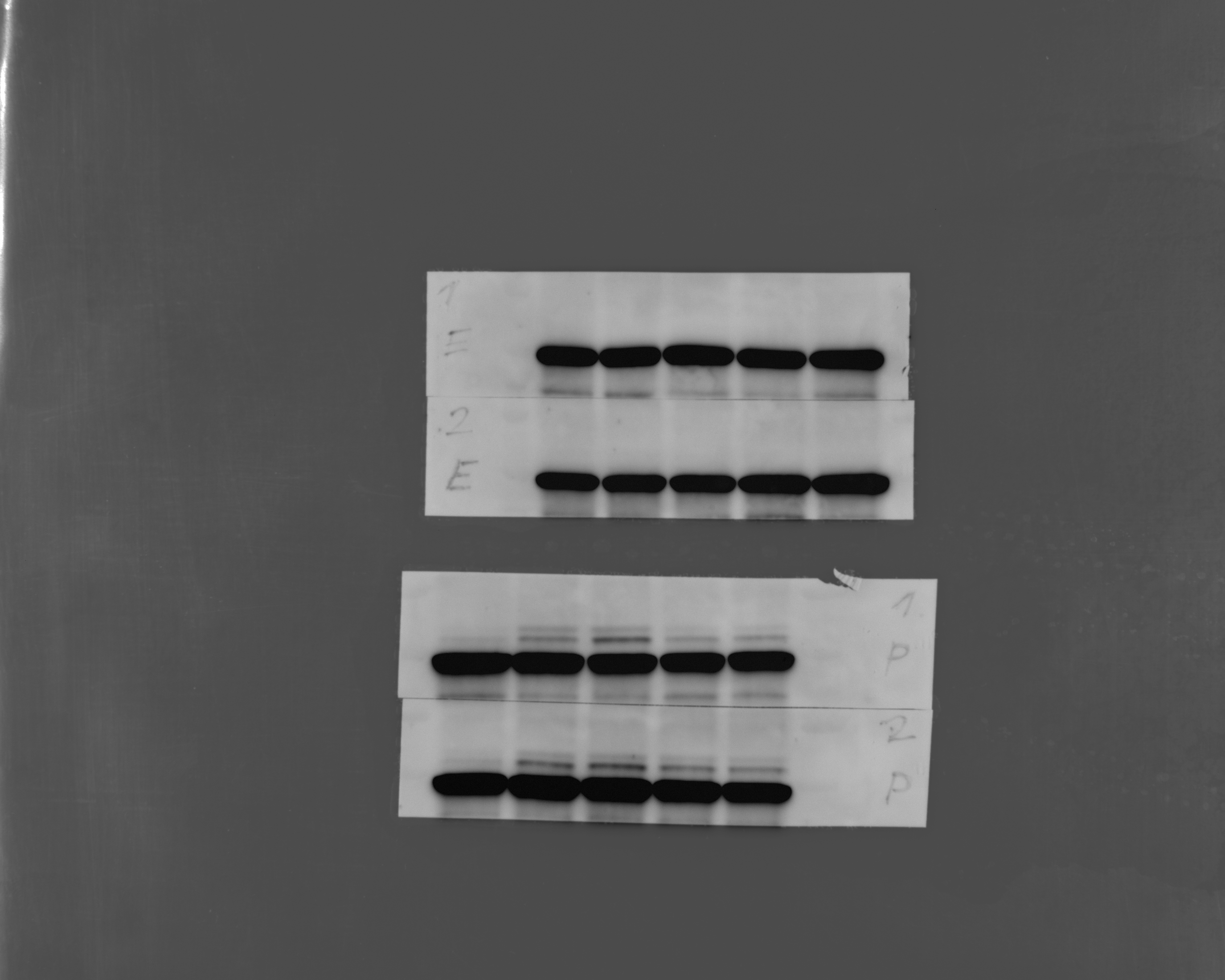

Supplement: Figure 5—source data 1. [file elife-95846-fig5-data1.zip › 5E/2023-05-05-100806 GAPDH reincubation.tif]

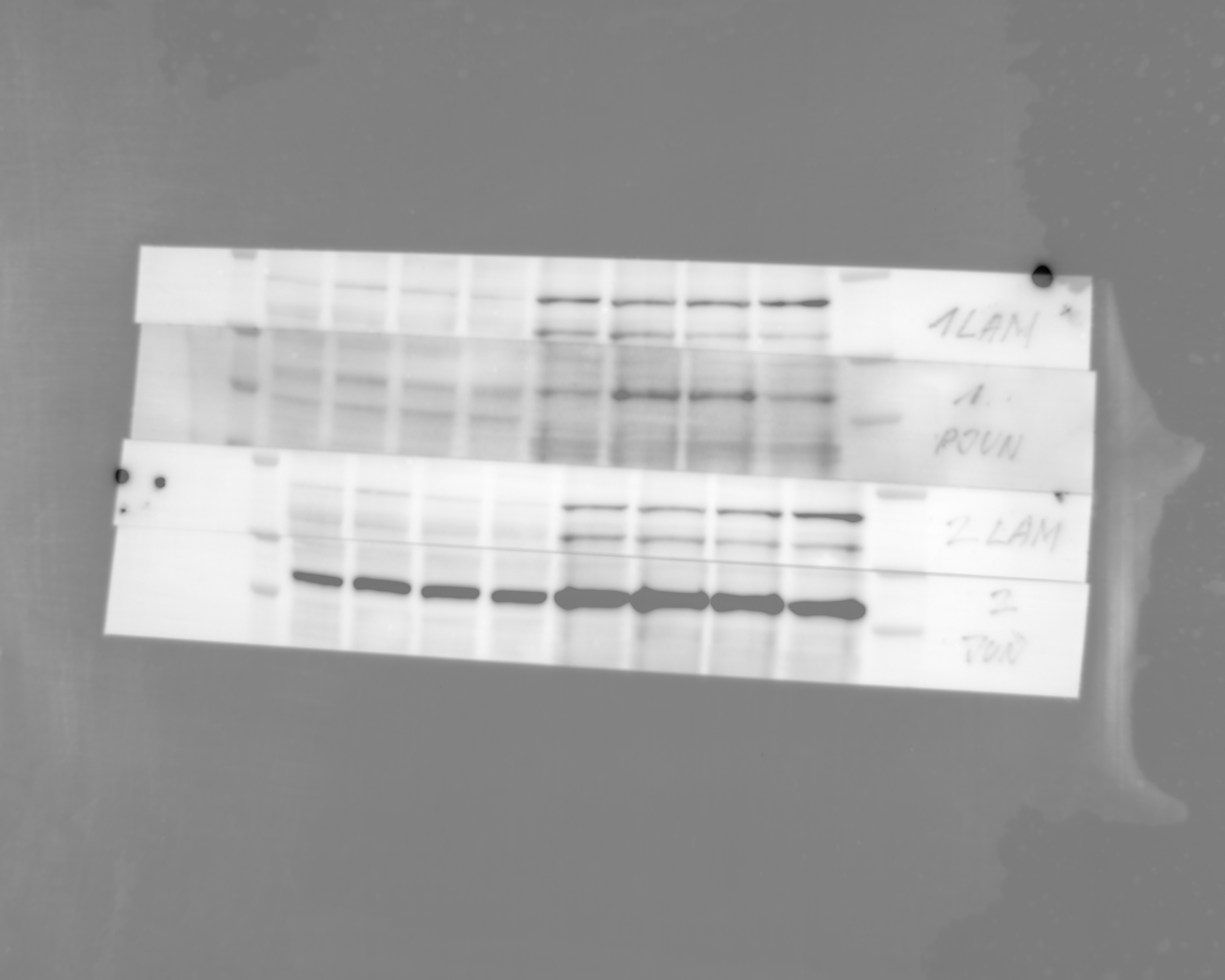

Supplement: Figure 5—source data 1. [file elife-95846-fig5-data1.zip › 5F/2024-05-03-111931 JUN, PJUN, LAMB1.tif]

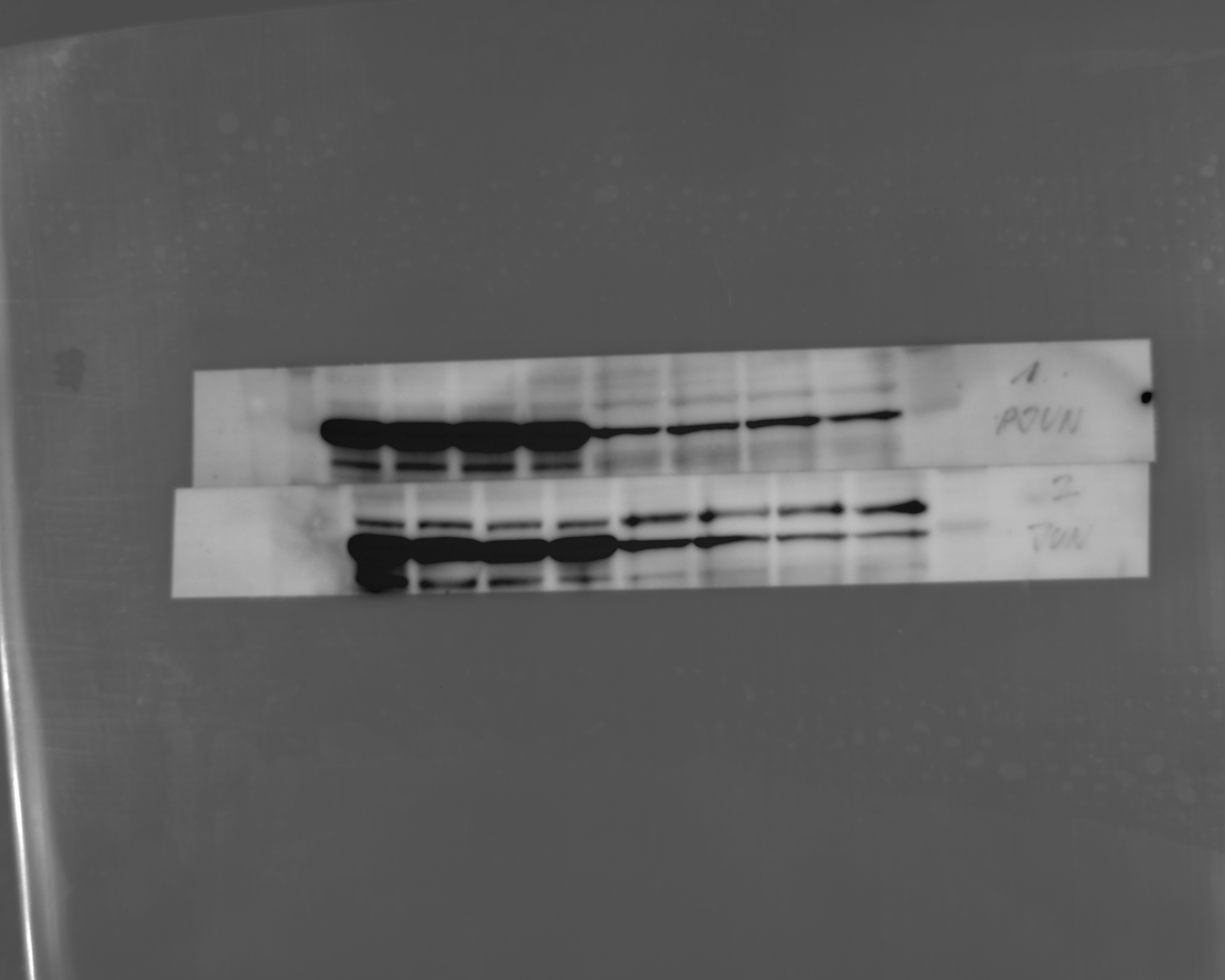

Supplement: Figure 5—source data 1. [file elife-95846-fig5-data1.zip › 5F/2024-05-07-110355 GAPDH reincubation.tif]

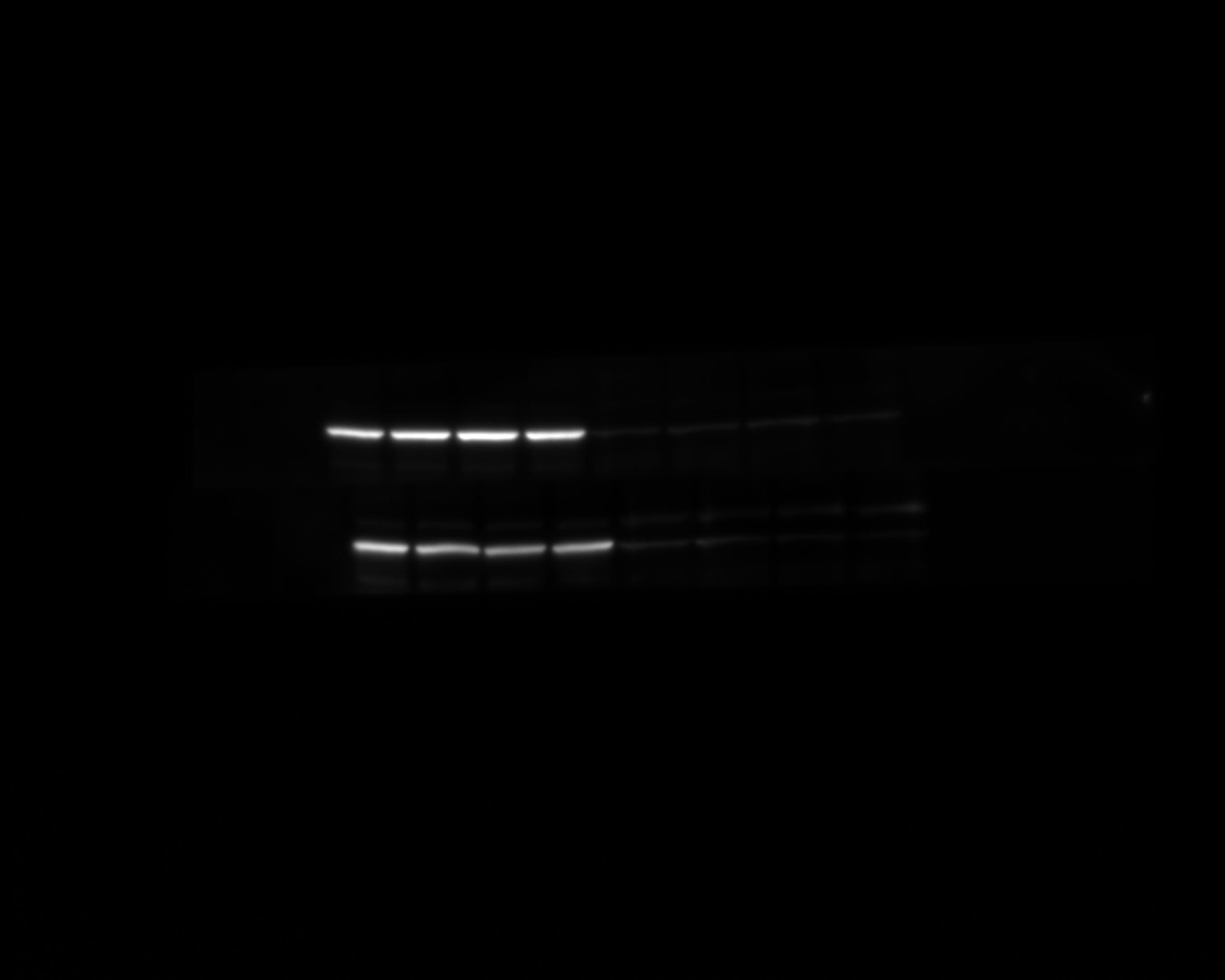

Supplement: Figure 5—source data 1. [file elife-95846-fig5-data1.zip › 5F/2024-05-07-110355-image1-sub2-GAPDH reincubation low exp.tif]

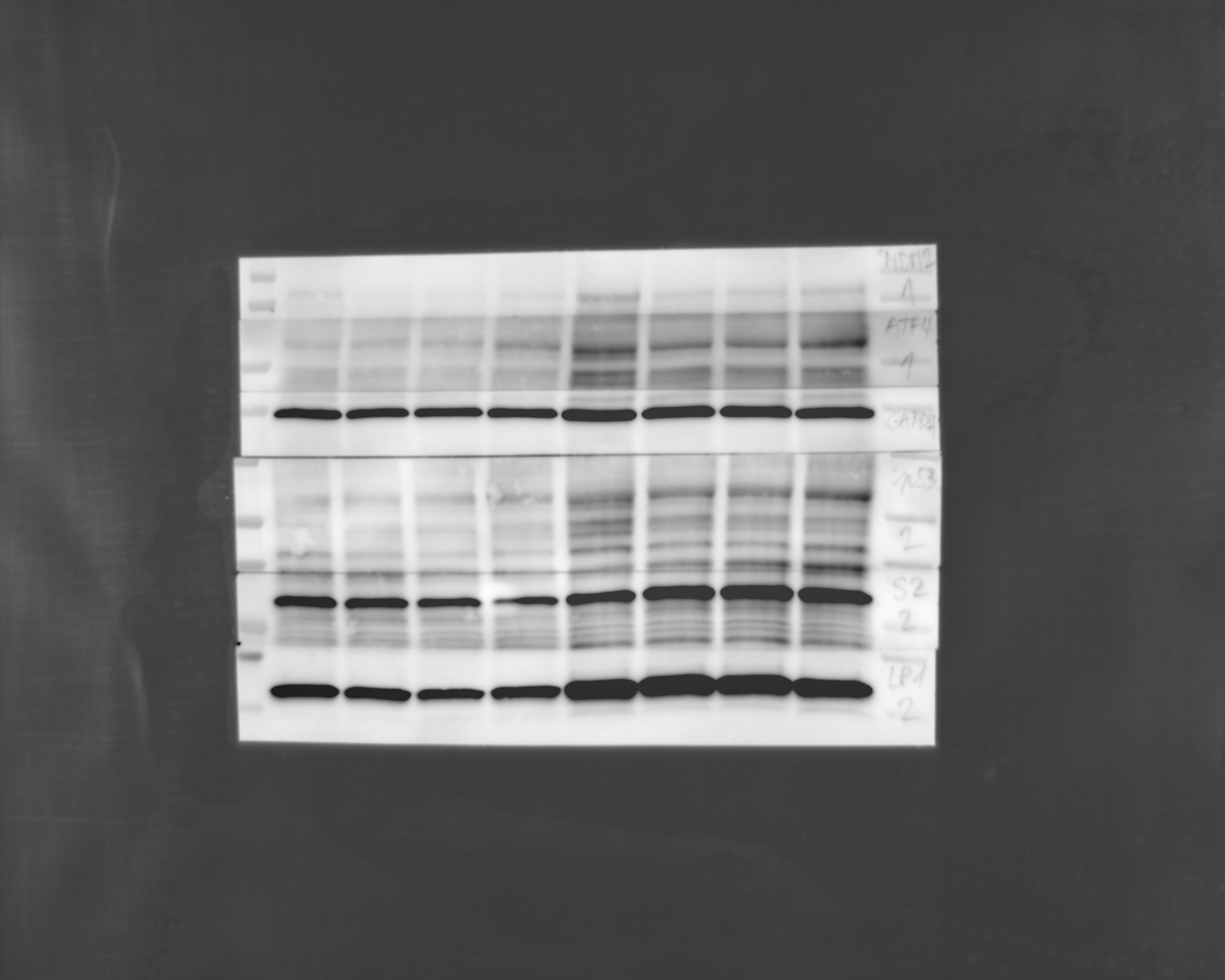

Supplement: Figure 6—source data 1. [file elife-95846-fig6-data1.zip › 2022-12-16-094318 MDM2, GAPDH.tif]

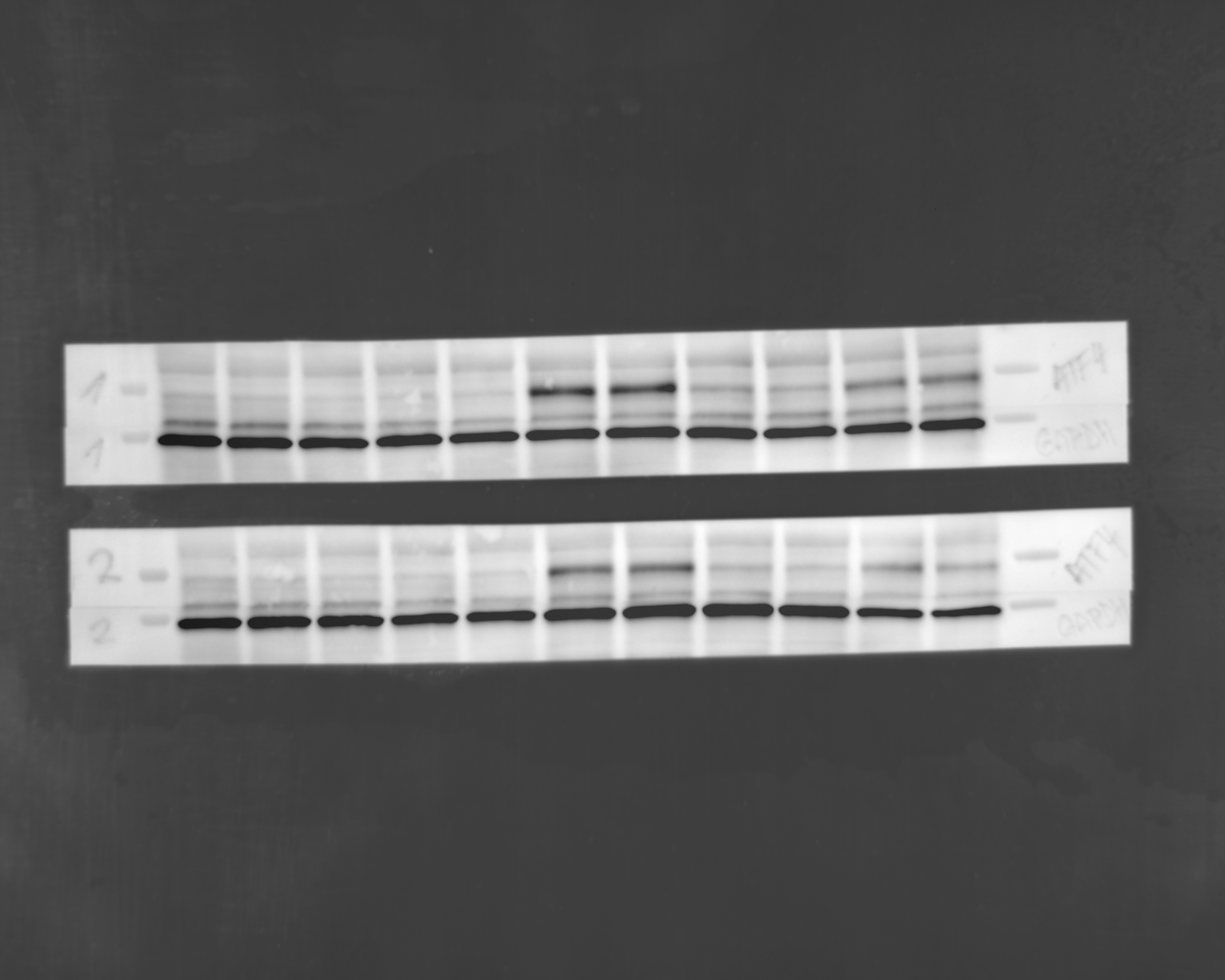

Supplement: Figure 6—source data 1. [file elife-95846-fig6-data1.zip › 2023-02-02-122453 ATF4, GAPDH.tif]

□

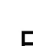

Supplement: Figure 6—source data 2. [file elife-95846-fig6-data2.pdf]
