# Supplementary material for: Perturbations in eIF3 subunit stoichiometry alter expression of ribosomal proteins and key components of the MAPK signaling pathways
Source: eLife. 2024 Nov 4;13:RP95846. doi: 10.7554/eLife.95846 (PMC11534336; doi:10.7554/eLife.95846)

Figure 1 - supplement 1C source data

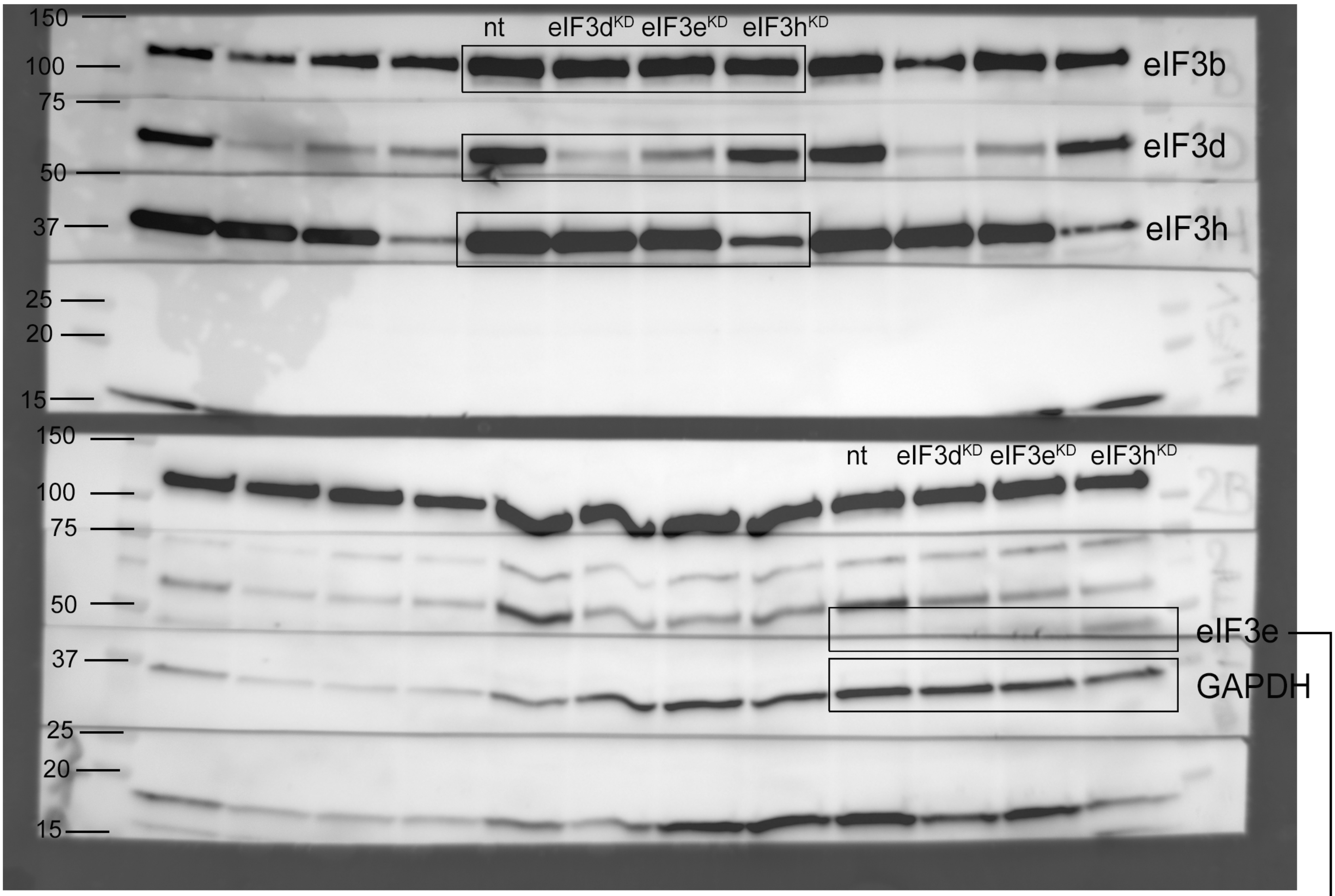

eIF3e strip was cut through so another technical replicate was run

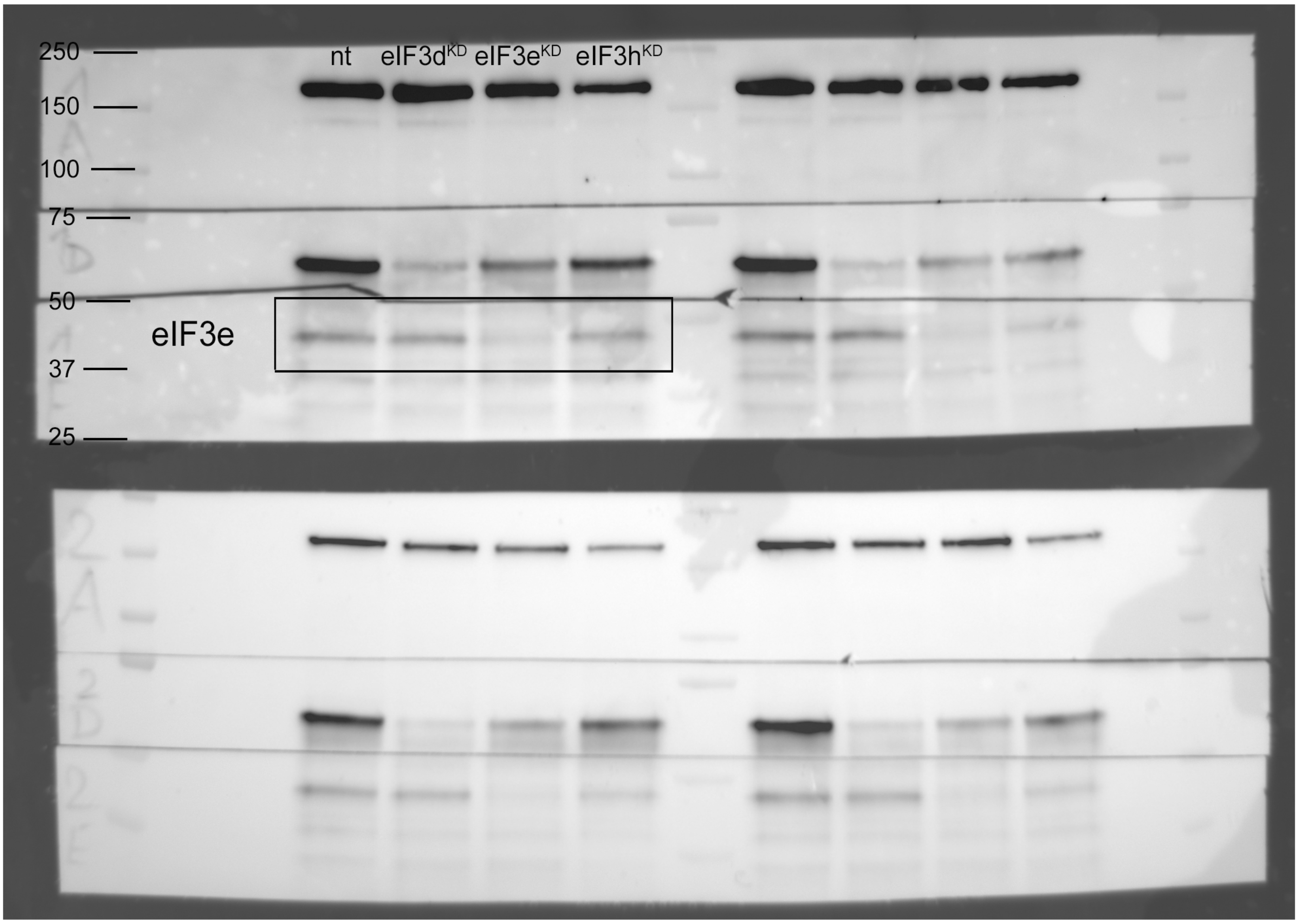

Supplement: Figure 1—figure supplement 1—source data 2. [file elife-95846-fig1-figsupp1-data2.pdf]
