# Supplementary material for: Perturbations in eIF3 subunit stoichiometry alter expression of ribosomal proteins and key components of the MAPK signaling pathways
Source: eLife. 2024 Nov 4;13:RP95846. doi: 10.7554/eLife.95846 (PMC11534336; doi:10.7554/eLife.95846)

Figure 5C source data

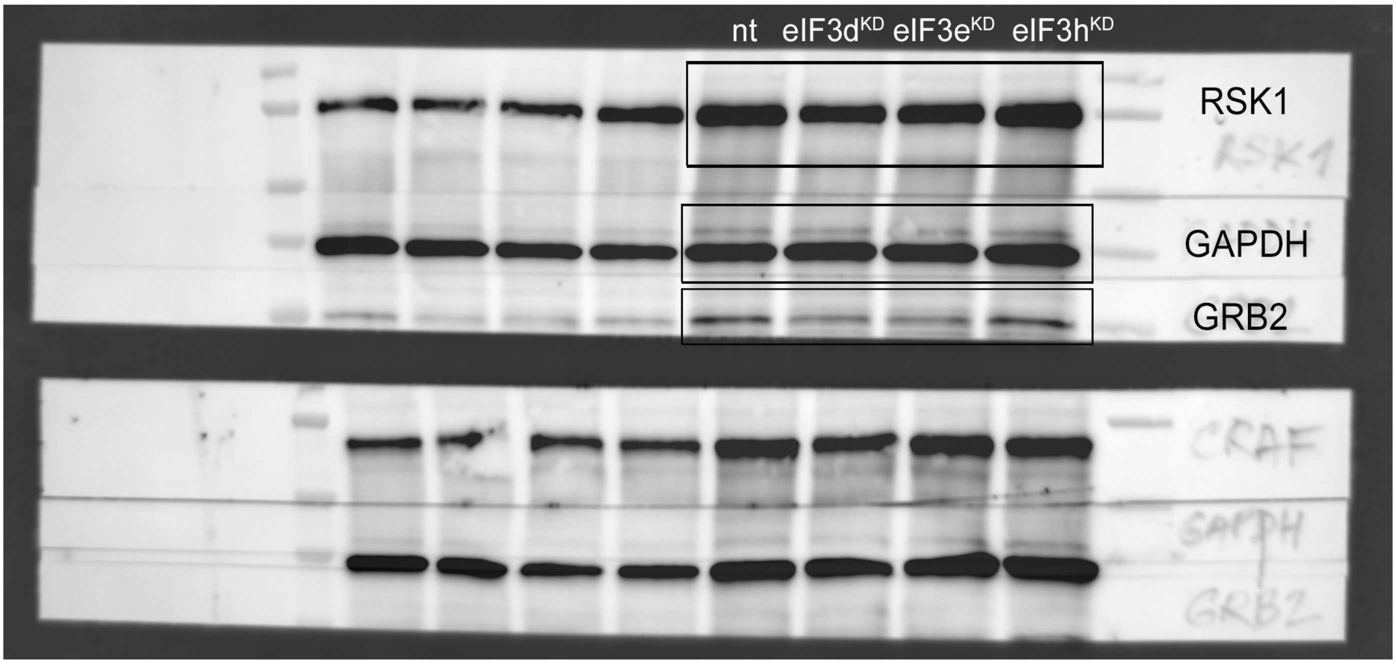

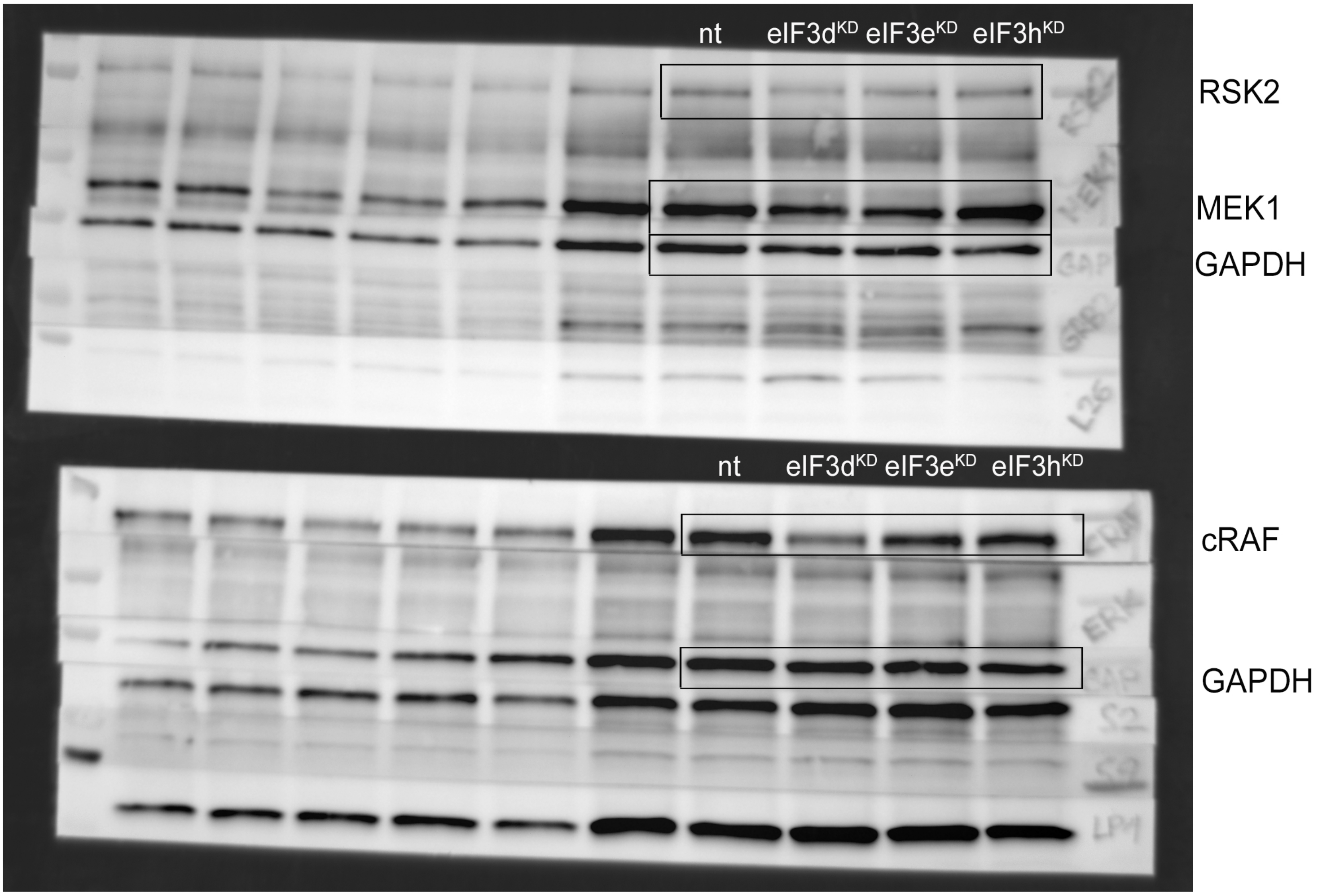

Figure 5C source data

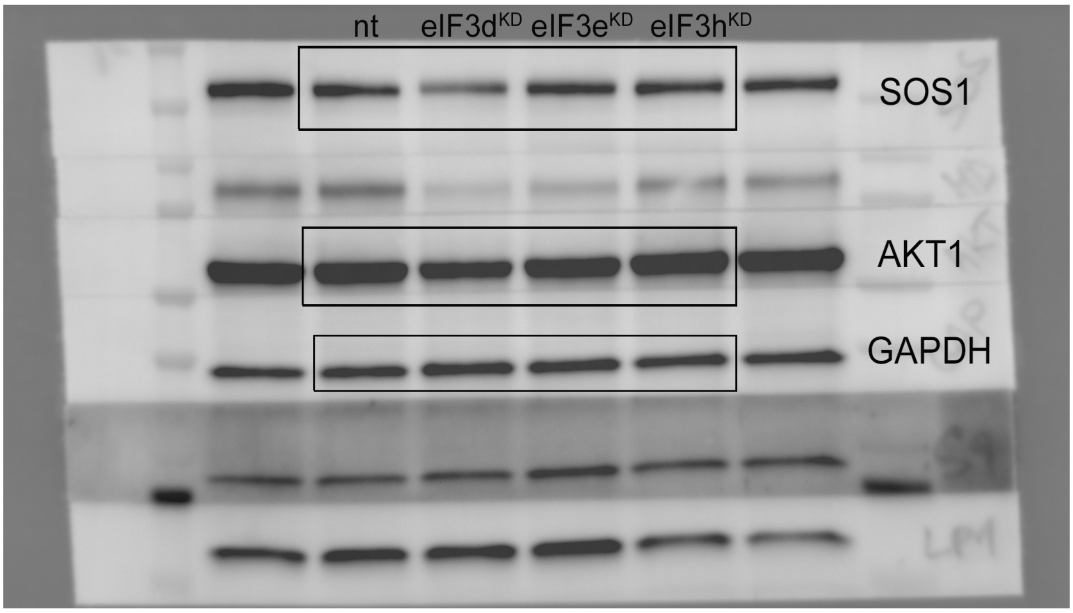

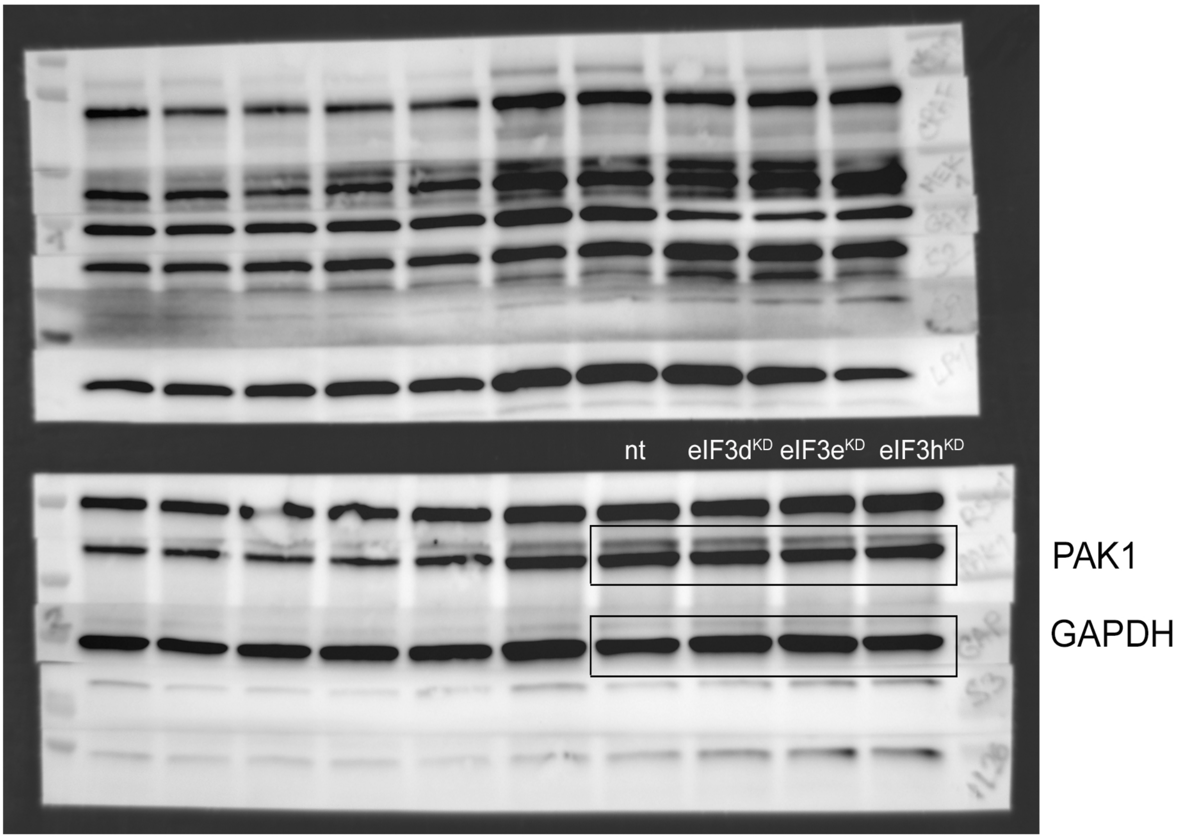

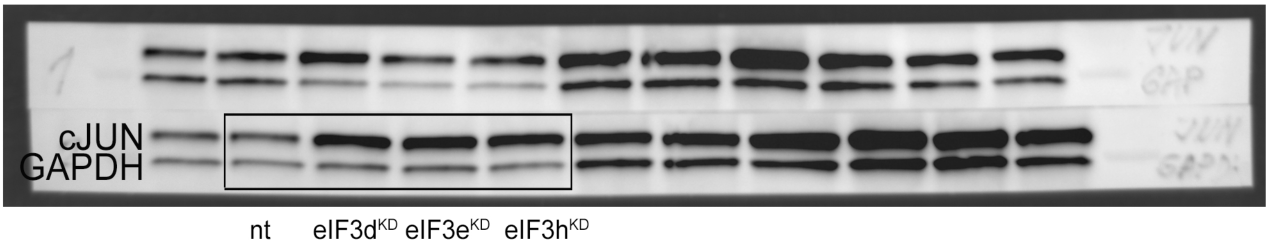

Figure 5E source data

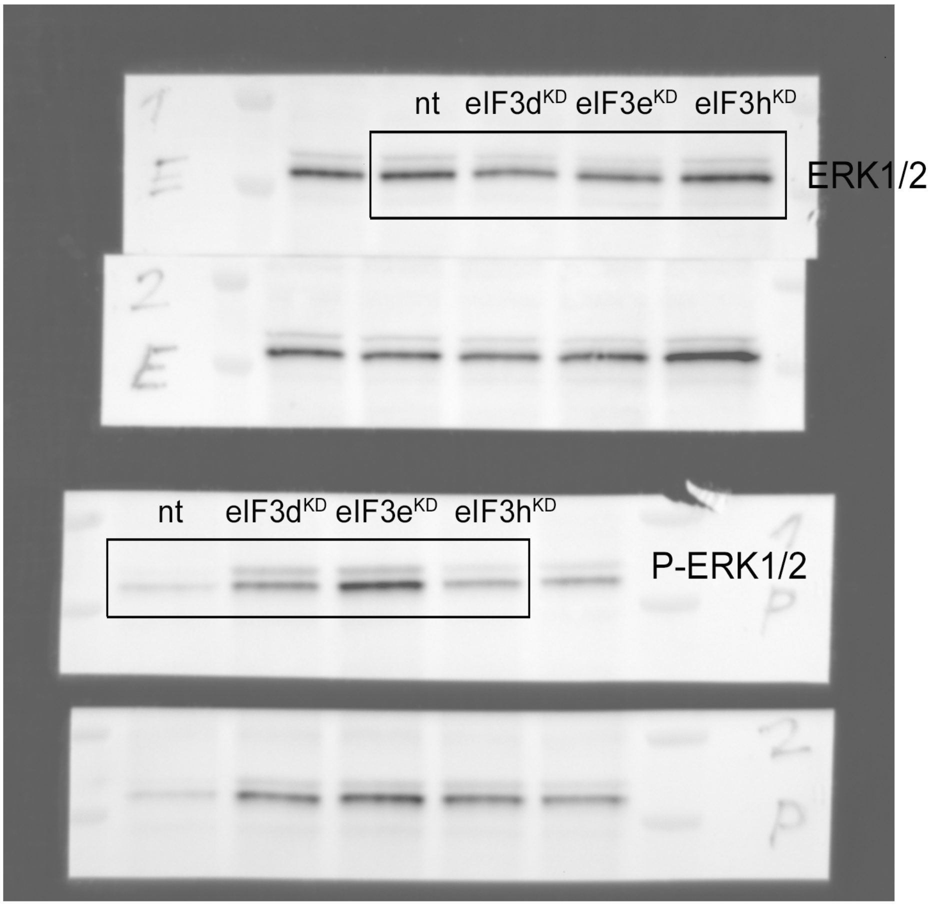

Reincubation of the same membrane to GAPDH

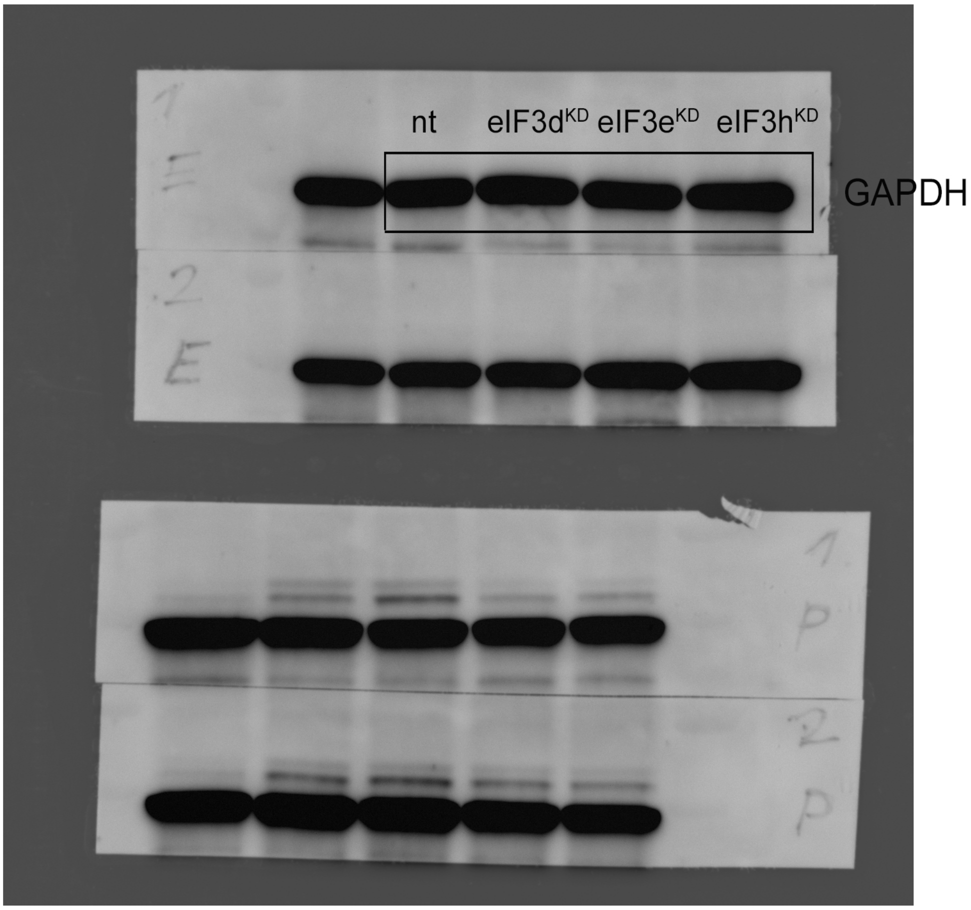

Figure 5F source data

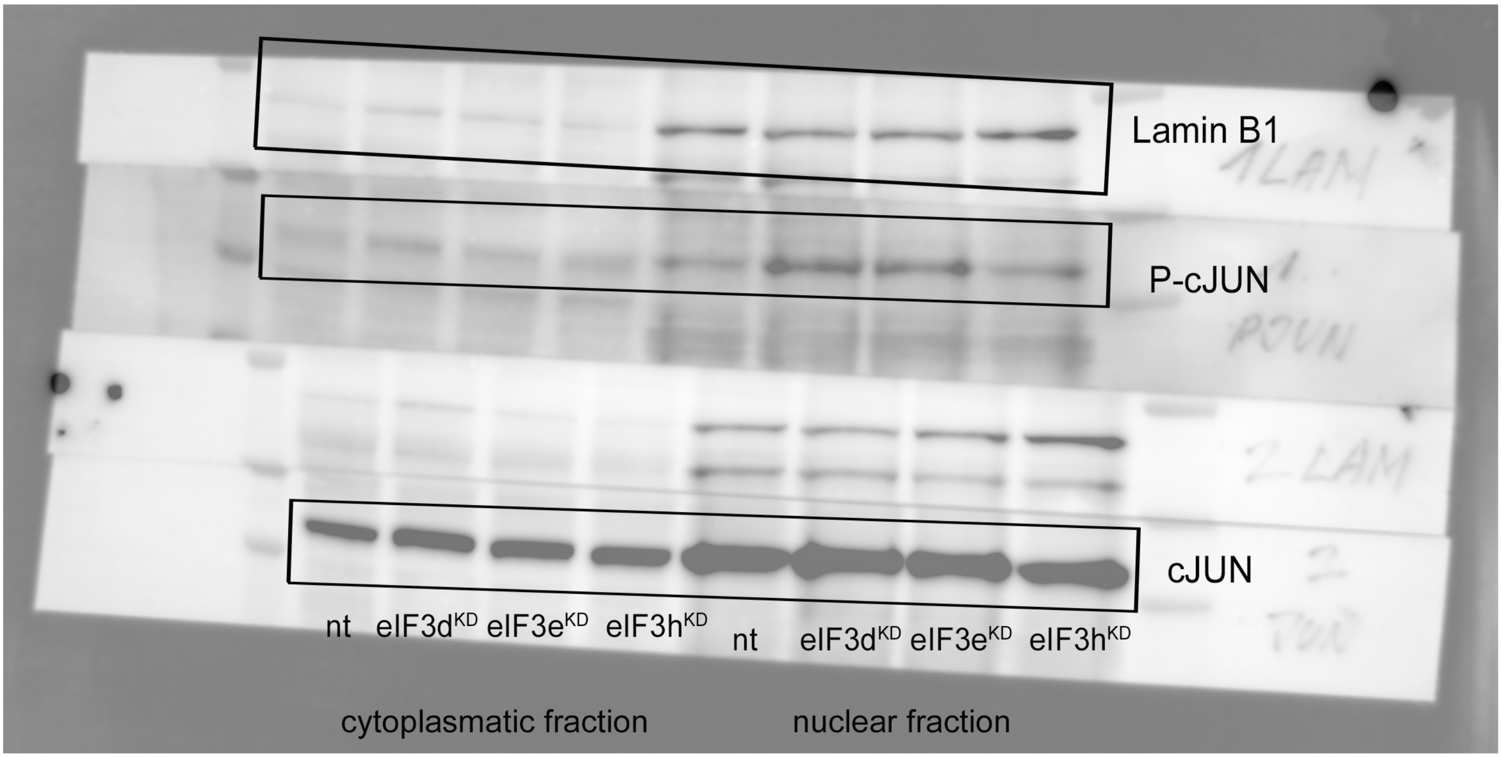

Reincubation of the same membrane to GAPDH

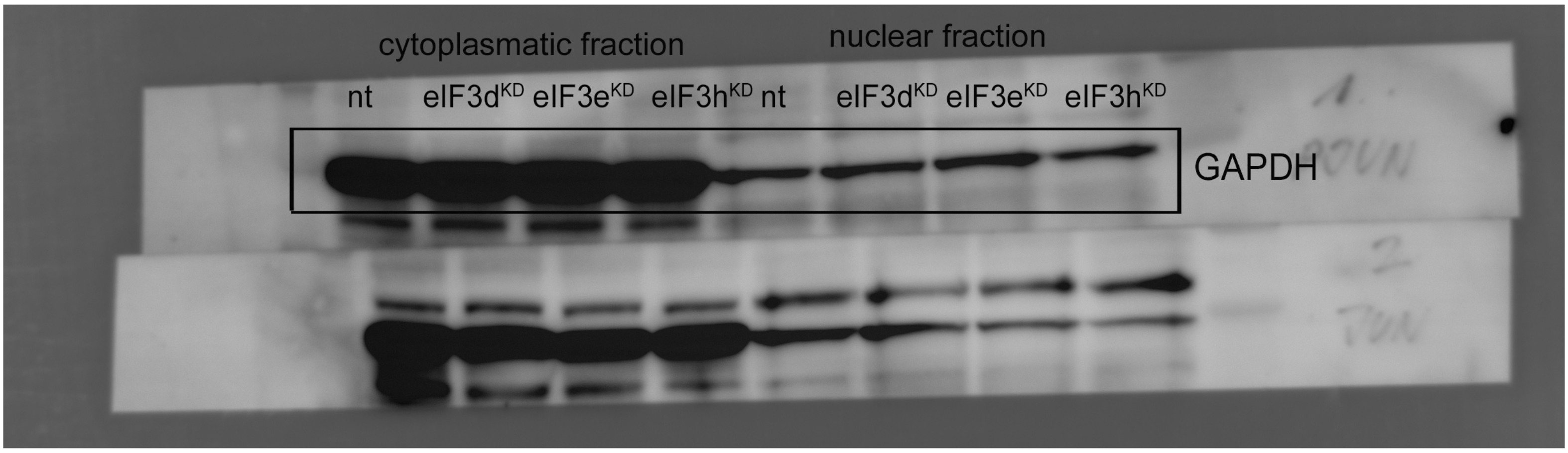

Reincubation of the same membrane to GAPDH - lower exposition of the same

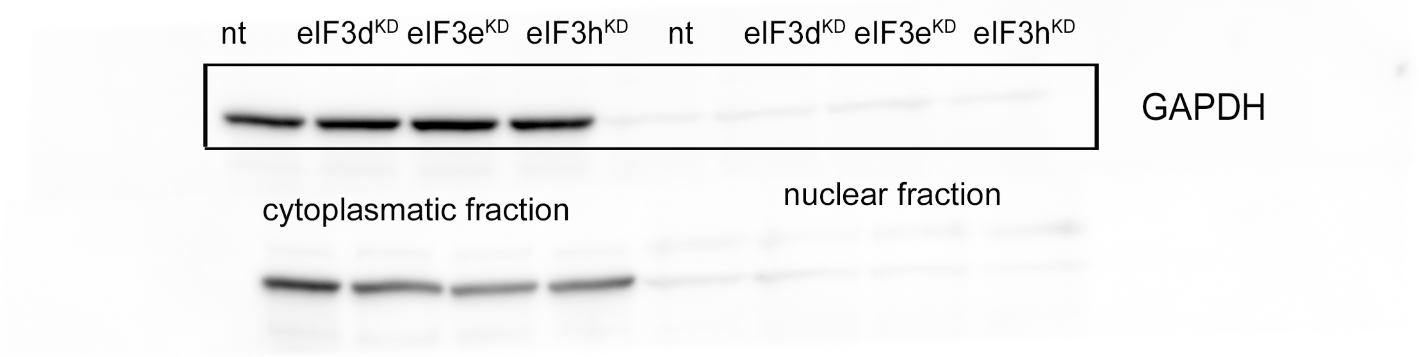

Supplement: Figure 5—source data 2. [file elife-95846-fig5-data2.pdf]
