# Supplementary material for: Perturbations in eIF3 subunit stoichiometry alter expression of ribosomal proteins and key components of the MAPK signaling pathways
Source: eLife. 2024 Nov 4;13:RP95846. doi: 10.7554/eLife.95846 (PMC11534336; doi:10.7554/eLife.95846)
Supplement: Supplementary file 2. [file elife-95846-supp2.docx]

**Supplementary Table A.** eIF3h^KD^ “all downregulated” DTEGs, top 8 significant results from KEGG pathways as evaluated by Enrichr.

| Term | p-value | Overlap genes |
| --- | --- | --- |
| Prostate cancer | 0.003773 | MDM2, ATF4 |
| Aldosterone synthesis and secretion | 0.003849 | PRKD3, ATF4 |
| Viral carcinogenesis | 0.015649 | MDM2, ATF4 |
| Human cytomegalovirus infection | 0.018997 | MDM2, ATF4 |
| Endocytosis | 0.023483 | MDM2, RAB11FIP2 |
| Bladder cancer | 0.038256 | MDM2 |
| PI3K-Akt signaling pathway | 0.043814 | MDM2, ATF4 |
| Cocaine addiction | 0.045558 | ATF4 |

**Supplementary Table B.** eIF3h^KD^ “all downregulated” DTEGs, top 10 significant results from GO Biological Process as evaluated by Enrichr.

| Term | p-value | Overlap genes |
| --- | --- | --- |
| Protein ubiquitination | 0.001329 | RNF10, RMND5A, WAC, MDM2 |
| Mitotic G1 DNA damage checkpoint signaling | 0.001716 | WAC, MDM2 |
| Protein autoubiquitination | 0.001716 | RNF10, MDM2 |
| Protein deubiquitination | 0.001946 | ZRANB1, USP38, MDM2 |
| Protein modification by small protein removal | 0.002139 | ZRANB1, USP38, MDM2 |
| Proteolysis involved in cellular protein catabolic process | 0.002217 | ZRANB1, MDM2 |
| RNA splicing | 0.003849 | IVNS1ABP, SNRPG |
| Regulation of proteasomal ubiquitin-dependent protein catabolic process | 0.004162 | WAC, MDM2 |
| Ubiquitin-dependent protein catabolic process | 0.004316 | ZRANB1, RMND5A, MDM2 |
| Protein K29-linked deubiquitination | 0.004741 | ZRANB1 |
