# Supplementary material for: Perturbations in eIF3 subunit stoichiometry alter expression of ribosomal proteins and key components of the MAPK signaling pathways
Source: eLife. 2024 Nov 4;13:RP95846. doi: 10.7554/eLife.95846 (PMC11534336; doi:10.7554/eLife.95846)
Supplement: Supplementary file 3. [file elife-95846-supp3.docx]

**Supplementary Table A.** siRNAs used in the study.

| **ON-TARGETplus siRNA DHARMACON** | **cat #** |
| --- | --- |
| eIF3d | L-017556-00 |
| eIF3e | L-010518-00 |
| eIF3h | L-003883-00 |
| Non-targeting | D-001810-03 |

**Supplementary Table B.** qPCR primers used in the study.

| **Name** | **5ˈto 3ˈ sequence** |
| --- | --- |
| Akt1* | CTTCTTTGCCGGTATCGTG |
| Akt1-R* | TGCTGTCATCTTGGTCAGG |
| ALAS1 | CCACTGGAAGAGCTGTGTGATGTG |
| ALAS1-R | GCGATGTACCCTCCAACACAACC |
| ATF4 | AAACCTTACGATCCTCCTGGAG |
| ATF4-R | TGGCTGCTGTCTTGTTTTGC |
| eIF3b* | TGTGAAAGGTACCTGGTGAC |
| eIF3b R* | AATAGGCCAATGGGCTGAG |
| eIF3d | CCAACCCAAACCCGTTTGTG |
| eIF3d R | TCTTCAGCTCCGTGGCAATG |
| eIF3e | CTGGTTCCAGCAACAGATAG |
| eIF3e R | GTGGCTGATAAAGGAAGAGG |
| eIF3h* | GCTGACTTTGATGAAGTCCA |
| eIF3h R* | ATGTGGACTGATACCAGCC |
| GRB2* | CTCTGTCAAGTTTGGAAACGA |
| GRB-R* | TGAACTTCACCACCCAGAG |
| Jun* | CAACATGCTCAGGGAACAG |
| Jun-R* | ACTGTTAACGTGGTTCATGAC |
| MAP2K1* | CCAGAAAGCTAATTCATCTGGAG |
| MAP2K1-R* | GTTGCACTCATGCAGAACC |
| PAK1* | CTTTGACCCGGAATACTGAG |
| PAK1-R* | CACTATGCTTCGTAATTTCTCC |
| RAF1* | CACAACTTTGCTCGGAAGAC |
| RAF1-R* | ACATCGAAATCCATTGAGCAG |
| RPL26* | GATGATGAAGTTCAGGTTGTACG |
| RPL26-R* | CATATTTCTTCCTGTAAACCTGGAC |
| RPLP1* | CTACTCGGCCCTCATTCTG |
| RPLP1-R* | CCACTTTCTTCTCCTCAGC |
| RPS2* | AGATCATTGATTTCTTCCTGGG |
| RPS2-R* | CAACAAATGCCTTGAACCTG |
| RPS3* | TTATCTTAGCCACCAGAACAC |
| RPS3-R* | TCTTCTGAACTACAGCAGTC |
| RPS6KA1* | AACGCTGAAAGTACGTGAC |
| RPS6KA1-R | CATAGTGCAGCTTCACCAC |
| RPS6KA3* | CTATACAATGCTTACCGGTTACAC |
| RPS6KA3-R* | CTACCTATTCGTGCCAATATTTCC |
| RPS9* | AAGAGCTGAAGCTGATCGG |
| RPS9-R* | AATTTGACCCTCCAGACCTC |
| SOS1* | TGGTGTCCTTGAGGTTGTC |
| SOS1-R* | GGCGACTTGGTATTTGCTC |

*primersˈ sequences were obtained from the database GETPrime, available at

http://bbcftools.epfl.ch/getprime

**Supplementary Table C.** Antibodies used in the study.

| **Antibody** | **source** |
| --- | --- |
| AKT1 | Cell Signaling Technology Cat# 2938, RRID:AB_915788 |
| ATF4 | Cell Signaling Technology Cat# 11815, RRID:AB_2616025 |
| eIF3b | Thermo Fisher Scientific Cat# PA5-23278, RRID:AB_2540802 |
| eIF3d | Atlas Antibodies Cat# HPA066216, RRID:AB_2685637 |
| eIF3e | Thermo Fisher Scientific Cat# PA5-29487, RRID:AB_2546963 |
| eIF3h | Cell Signaling Technology Cat# 3413, RRID:AB_2277726 |
| ERK1/2 | Santa Cruz Biotechnology Cat# sc-514302, RRID:AB_2571739 |
| P-ERK1/2 | Cell Signaling Technology Cat# 4370, RRID:AB_2315112 |
| GAPDH | Thermo Fisher Scientific Cat# PA1-987, RRID:AB_2107311 |
| GRB2 | BD Biosciences Cat# 610112, RRID:AB_397518 |
| JUN | Cell Signaling Technology Cat# 9165, RRID:AB_2130165 |
| P-JUN | Cell Signaling Technology Cat# 9261, RRID:AB_2130162 |
| LAMIN B1 | Cell Signaling Technology Cat# 12586, RRID:AB_2650517 |
| MDM2 | Cell Signaling Technology Cat# 86934, RRID:AB_2784534 |
| MEK1 | BD Biosciences Cat# 610122, RRID:AB_397528 |
| PAK1 | Santa Cruz Biotechnology Cat# sc-881, RRID:AB_2160720 |
| RAF | Santa Cruz Biotechnology Cat# sc-133, RRID:AB_632305 |
| RPL13A | Cell Signaling Technology Cat# 2765, RRID:AB_916223 |
| RPL26 | Cell Signaling Technology Cat# 5400, RRID:AB_10698750 |
| RPLP1 | Sigma-Aldrich Cat# HPA003368, RRID:AB_1079845 |
| RPS2 | Santa Cruz Biotechnology Cat# sc-130399, RRID:AB_2238506 |
| RPS3 | Proteintech Cat# 15198-1-AP, RRID:AB_2253905 |
| RPS9 | Thermo Fisher Scientific Cat# PA5-13569, RRID:AB_2182393 |
| RSK1 | Santa Cruz Biotechnology Cat# sc-231, RRID:AB_632367 |
| RSK2 | Santa Cruz Biotechnology Cat# sc-9986, RRID:AB_672176 |
| SOS1 | Cell Signaling Technology Cat# 12409, RRID:AB_2797902 |
